# Ionic Conductive Textiles for Wearable Technology

**DOI:** 10.1002/adma.202502140

**Published:** 2025-06-05

**Authors:** Lingtao Fang, Yunlu Zhou, Qiyao Huang

**Affiliations:** ^1^ School of Fashion and Textiles The Hong Kong Polytechnic University Hong Kong SAR 999077 China; ^2^ Research Institute for Intelligent Wearable Systems The Hong Kong Polytechnic University Hong Kong SAR 999077 China

**Keywords:** ionic conductive textiles, ionic conductors, wearable technologies

## Abstract

Soft ionic conductors, characterized by their inherent flexibility and tissue‐like ion dynamics, are ideal for intimate applications such as wearable electronics for sensing, energy harvesting, signal transmission, and bioelectronics applications. Shaping ionic conductors into fiber and textile formats (i.e., ionic conductive textiles) to replace the focus on rigid electron‐based conductors heralds a transformative technology in wearable electronics and smart textiles, offering advantages that align with human‐device compatibility, wearability, and sustainability demands. In this review, the category of ionic conductors, the essential characteristics of ionic conductors, the methodologies for the fabrication and integration of ionic conductive textiles, and the diverse applications of these textile‐based soft ionic conductors are summarized. By providing perspectives and raising potential challenges on the future design and development of ionic conductive textiles in terms of sustainability, wearability, fabrication strategies, and integration with electrical systems, this review aims to highlight the potential of ionic conductors as key components for the next generation of wearable technologies and electronic textiles.

## Graphical abstract

Recent advances in ionic conductive textiles for wearable technology are summarized, with a focus on soft ionic conductors that exhibit skin‐like flexibility and tissue‐like ion dynamics. Their structures, key characteristics, manufacturing methods, and diverse applications are reviewed. Future challenges in sustainability, wearability, fabrication, and electronic integration are discussed, highlighting their potential in healthcare and interactive clothing.

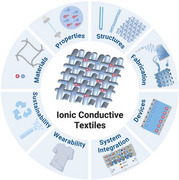

## 1 Introduction

The rapid development of electronic textiles (E‐textiles) has revolutionized the integration of electronic devices into wearable formats, allowing the electronics to be worn against the skin akin to textiles for diverse applications ranging from healthcare monitoring, therapy, and rehabilitation, to soft robotics.^[^ ^1^, ^2^, ^3^, ^4^, ^5^, ^6^ ^]^ The electrical functionalities of these novel soft electronics mostly rely on electrically conductive textiles, such as metallic textiles, carbon fibers, and conducting polymer‐coated fabrics, which are the electrical conductors that realize the electric signal transfer through electron movement within the device.^[^ ^7^, ^8^, ^9^ ^]^ Owning to the multi‐dimensional fiber‐yarn‐fabric structures as well as the exceptional electrical conductivity of these conductive textiles, cutting‐edge E‐textiles have illustrated the feasibility of developing smart garments and wearable accessories that integrated with 1D fiber‐shaped or 2D fabric‐shaped sensors, energy harvesting units, capacitors and batteries, and communication devices. Such a seamless integration showcases the vast potential of these intelligent E‐textile technologies in the field of future wearable technology.^[^ ^10^, ^11^, ^12^, ^13^ ^]^ Despite their compatibility with existing wearable technologies, electron‐based conductive textiles have difficulties meeting demands such as transparency in optoelectronic devices and biocompatibility in skin‐attachable bioelectronics.^[^ ^14^ ^]^ On the other hand, human skin and tissues rely on ions as charge carriers to transmit signals in response to various stimuli. Such a mechanism mismatch in the signal transmission between the electron‐based conduction in electrically conductive textiles and the ion‐based signaling in biological systems makes device‐skin compatibility and bio‐signal detection for E‐textiles challenging.^[^ ^15^ ^]^

**Figure 1:**
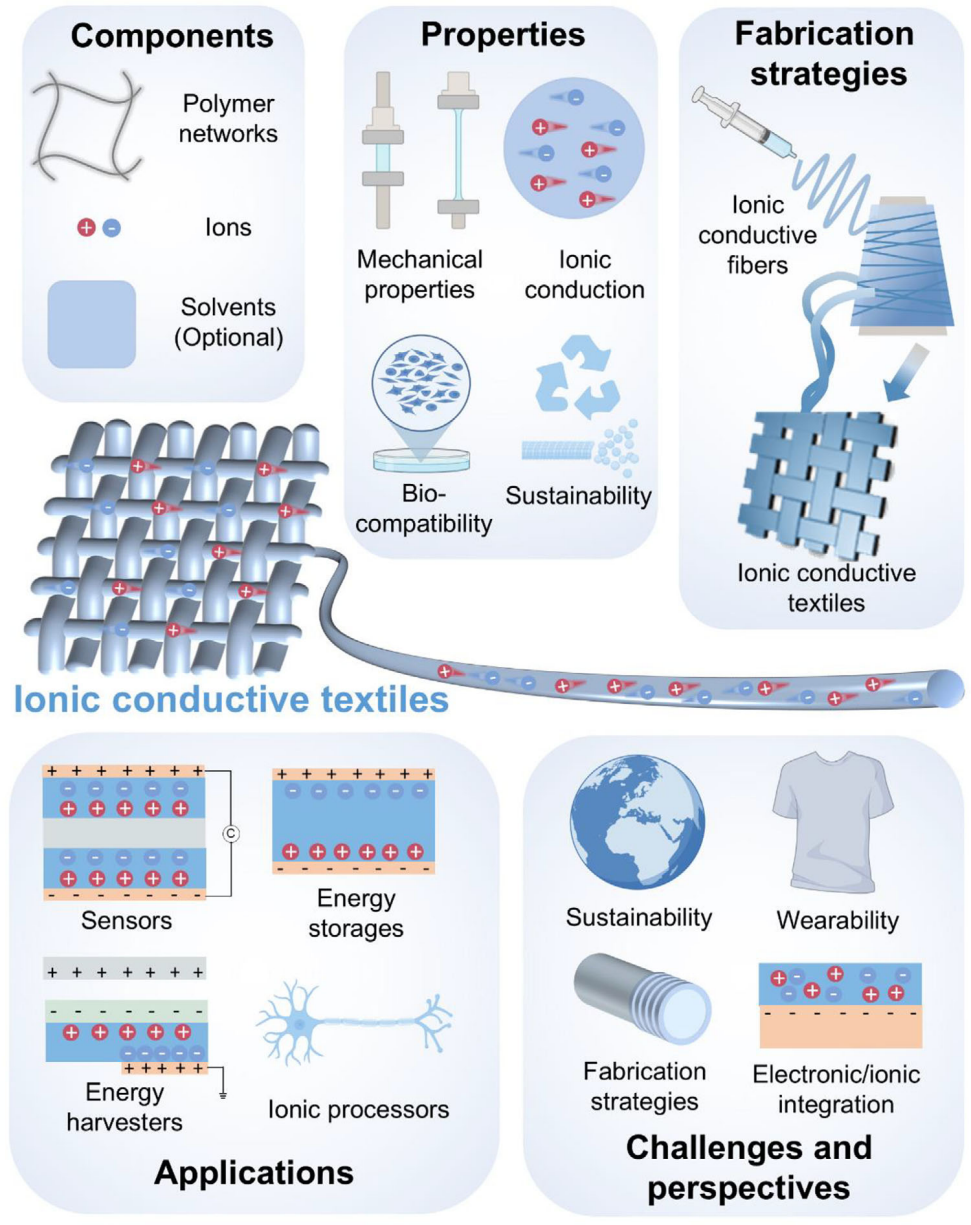
Structure of this review, including components and properties of textile‐based ionic conductors, fabrication strategies of ionic conductive fibers/fabrics, applications, as well as challenges and perspectives. The icons corresponding to mechanical properties, biocompatibility, sustainability, ionic processors, and wearability were created with BioRender.com.

Ionotronics, focusing on ion‐based devices that are realized by utilizing ionic conductors as the building blocks, have emerged as a crucial area of wearable devices in response to the abovementioned challenges, particularly in biomedical applications.^[^ ^16^, ^17^, ^18^, ^19^, ^20^ ^]^ Over the past decade, numerous ionic conductors, such as hydrogels, ionogels, and organohydrogels, composed of polymeric networks swollen with diverse solvents, have been explored and employed in ionotronic applications.^[^ ^21^, ^22^, ^23^, ^24^ ^]^ They offer intrinsic flexibility and stretchability, tunable modulus, conductivity, and transparency, thereby making them a promising alternative to rigid and opaque E‐textiles for a more conformable interface between humans and wearable devices.^[^ ^25^, ^26^, ^27^ ^]^ Nevertheless, most ionic conductors for ionotronics are developed into 2D thin films or 3D bulky composites, largely limiting their ability to conform to irregular surfaces, especially human skin at joints (e.g., fingers and elbows). Shaping them into 1D fibers or 2D fibrous textile formats can endow the ionic conductors with excellent compliance on various geometries and good permeability for long‐term wearing.^[^ ^28^ ^]^ Therefore, ionic conductive textiles have been explored as alternatives to electron‐based conductive textiles for wearable E‐textiles.^[^ ^29^ ^]^ Despite the substantial studies on ionic conductors, there is a lack of reviews specifically focusing on textile‐based ionic conductors and ionotronics for wearable systems. In this review, we aim to fill this gap by summarizing the development of ionic conductive textiles (**Figure** **1**). We first classify the types of stretchable ionic conductors, providing a detailed understanding of their unique characteristics and properties. Additionally, representative strategies employed to regulate the properties of ionic materials, such as improving mechanical strength, enhancing ionic conductivity, and optimizing biocompatibility and sustainability, are introduced. Furthermore, the fiber spinning strategies, yarn engineering methods, and textile manufacturing techniques are summarized in detail, elucidating the key processes and mechanisms involved in fabricating high‐quality ionic conductive fibers and textiles. Importantly, we provide an overview of the application of ionic conductive textiles in sensors, energy harvesters, energy storage, and ionic processors. Lastly, we suggest future directions for the design of ionic conductive textiles from the perspectives spanning sustainability, wearability, fabrication strategies, and integration with electrical systems.

## 2 Classification of Ionic Conductors

**Figure 2:**
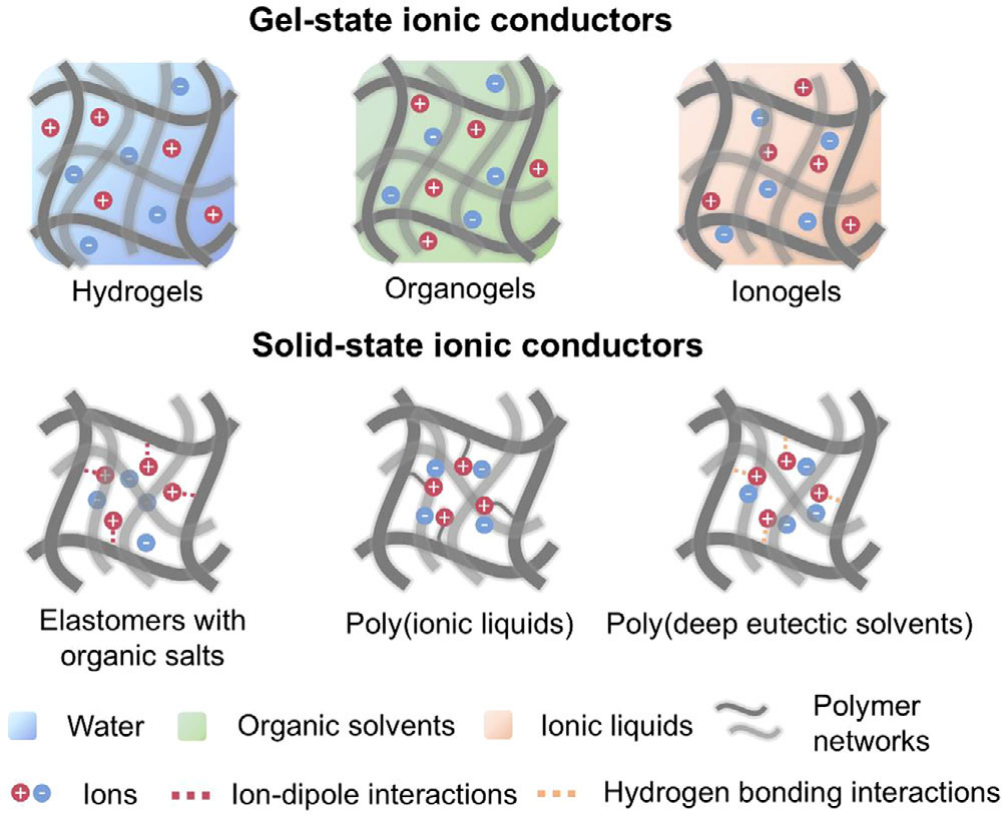
Categories and structures of gel‐state and solid‐state ionic conductors.

Ionic conductors mainly comprise polymer networks to maintain their shape and ions to achieve conductivity. They are typically formed by polymerizing monomers or soaking polymers in ion‐containing solvents. Ionic conductors can be divided into four types: hydrogels, organogels, ionogels, and ionoelastomers (**Figure** **2**). Hydrogels, organogels, and ionogels are termed gel‐state ionic conductors containing solvents (e.g., water, organic solvents, and ionic liquids), where ions are freely dissociated and in random movement within the solution. Ionoelastomers can be classified as solid‐state ionic conductors. This is because ionoelastomers consist of liquid‐free but ion‐containing polymer networks (e.g., elastomers with organic salts, poly(ionic liquids), and poly(deep eutectic solvents)), where ions are confined or bound to the polymer networks. Different types of ionic conductors exhibit unique characteristics. In general, gel‐state ionic conductors demonstrate high ionic conductivity (typically above 0.1 S m^−1^) but are sensitive to moisture and temperature variations caused by solvent evaporation and absorption. Among the gel‐state category, hydrogels are known for their exceptionally high stretchability and biocompatibility due to the high water content (60–90%). By incorporating ions hydrated with water, the hydrogels exhibit excellent anti‐freezing properties under −40 °C. Organogels and ionogels possess excellent anti‐dehydration and anti‐freezing properties due to the utilization of low‐volatile solvents (i.e., organic solvents for organogels and ionic liquids for ionogels) that exhibit higher thermal and chemical stability, while the mobility of solvents leads to the liquid leakage. In contrast, solid‐state ionic conductors exhibit high stabilities under cyclic load‐unloading (up to 100 000 cycles) and high temperature (up to 100 °C) characterized by the absence of liquid leakage, a wide electrochemical window, excellent waterproofness, and non‐volatility; however, they are limited by their low ionic conductivity (10^−5^–10^−3^ S m^−1^). Detailed structural characteristics and properties of each type of ionic conductor are discussed in this session, accompanied by examples presented in **Table** **1**.

**Table 1:** Examples of components and ionic conductivity of gel‐state and solid‐state ionic conductors.

Classification		Polymer matrix^a)^	Conducting salts^b)^	Solvents^c)^	Strength [MPa]	Stretchability [%]	Ionic conductivity [S m^−1^]	Stability	Refs.
Gel‐state ionic conductor	Hydrogels	PAM	LiCl	H_2_O	0.09–0.25	731–1099	2.25	−40–25 °C	[30]
		PAM	ZnCl_2_		35–180	50–500	7.14	−20–25 °C	[31]
		PAANa	Na^+^		5–7	600–1950	2	−35–20 °C	[32]
	Organogels	PAM/Nanoclay	Na^+^	EG/H_2_O	0.09	700	≈0.01	−30–40 °C	[33]
		Gelatin	Na_3_Cit	Gly/H_2_O	0.2–1.91	214–542	0.47	−60–20 °C	[34]
		PVA/CNFs	NaCl	DMSO/H_2_O	0.6–2.1	400–630	3.2	−70–25 °C	[35]
	Ionogels	PVA/PAA	[EMIm][DCA]	[EMIM][DCA]	1–1.5	2600	3.18	−30–25 °C	[36]
		PAMPS	[EMIm][DCA]	[EMIm][DCA]	0.4	158	1.9	−80–100 °C	[37]
		PU	[DEIM][TFSI]	[DEIM][TFSI]	1.56–2.52	215–327	0.12	Stored in the open air for 200 days, 10000 strain cycles	[38]
Solid‐state ionic conductor	Ionoelastomers with organic salts	P(IBA‐co‐MEA)	LiTFSI		1.05‐7.12	1400–1744	5.28 × 10^−3^	10–80 °C	[39]
		P(HFBA‐co‐OEGA)	LiTFSI		0.367–0.77	3155–6000	3.5 × 10^−3^	0–100 °C, waterproof	[40]
	Poly(ionic liquids)	P(AMT‐co‐MA)‐PMA	TFSI^−^		0.2–0.56	30–61.3	2.47 × 10^−5^	100000 strain cycles	[41]
		P(AA‐co‐VEIM)	Br^−^		0.025–0.23	260–750	1.74	waterproof	[42]
	Poly(deep eutectic solvents)	PAA	ChCl/LiCl		0.6–15	300–550	6 × 10^−3^	20–80 °C	[43]
		P(HFBA‐co‐IBOA)	N_444_BF_4_		0.16–0.65	900–1800	0.12	waterproof	[44]

^a)^ PAM: polyacrylamide, PAANa: sodium polyacrylate, PVA: polyvinyl Alcohol, CNFs: celluouse nanofibers, PAA: polyacrylic acid, PAMPS: poly(2‐acrylamido‐2‐methyl‐1‐propanesulfonic acid), PU: polyurethane, P(IBA‐co‐MEA): poly(isobornyl acrylate‐co‐ethylene glycol methyl ether acrylate), P(HFBA‐co‐OEGA): poly(2,2,3,4,4,4‐hexafluorobutyl acrylate)‐random‐poly(oligoethylene glycol methyl ether acrylate), P(AMT‐co‐MA)‐PMA: poly(1‐(3‐(acryloyloxy)propyl)‐3‐methylimidazolium bis(trifluoromethane)sulfonimide)‐co‐methyl acrylate)‐polymethyl acrylate, P(AA‐co‐VEIM): poly(acrylic acid‐co‐1‐vinyl‐3‐ethylImidazolium), P(HFBA‐co‐IBOA): poly(2,2,3,4,4,4‐hexafluorobutyl acrylate‐co‐isobornyl acrylate);

^b)^ Na_3_Cit: sodium citrate, [EMIm][DCA]: 1‐ethyl‐3‐methylimid‐azolium dicyanamide, [DEIM][TFSI]: 1,2‐dimethyl‐3‐ethoxyethyl‐imidazolium bis(trifluoromethanesulfonyl)imide, LiTFSI: Lithium bistrifluoromethane sulfonimide, ChCl: choline chloride, N_444_BF_4_: tetrabutylammonium tetrafluoroborate;

^c)^ EG: ethylene glycol, Gly: glycerol, DMSO: dimethylsulfoxide.

### 2.1 Gel‐State Ionic Conductors

Gel‐state ionic conductors consist of polymer networks, conductive ions, and solvents. Based on the diverse types of solvents, gels can be classified into hydrogels (with water as solvents), organogels (employing organic solvents), and ionogels (utilizing ionic liquids as solvents). In this section, the characteristics of the gels are summarized with examples of fiber‐based gel‐state ionic conductors.

#### 2.1.1 Hydrogel‐Based Ionic Conductors

Ionic conductive hydrogels, composed of hydrophilic polymer networks, water, and ions, have been extensively investigated owing to their remarkable properties, including softness, good stretchability, high transparency, and excellent ionic conductivity. The polymer networks function as an elastic framework, endowing the hydrogels with mechanical stretchability. Water, which functions as the solvent in the matrix of the hydrogel, plays a crucial role as a plasticizer, enhancing the mobility of both polymer chains and ions. The increased mobility and porous structure not only ensure the deformability of the hydrogels, allowing them to withstand significant mechanical deformation without rupturing but also enhance their ionic conductivity. Meanwhile, the ions provide conductivity, enabling efficient charge transport and transduction of biological signals within the hydrogel matrix.^[^ ^45^ ^]^

Two methods are widely used to introduce ions into hydrogel systems. One is dissolving various salts, such as lithium chloride (LiCl), sodium chloride (NaCl), potassium chloride (KCl), zinc chloride (ZnCl_2_), magnesium chloride (MgCl_2_), aluminium chloride (AlCl_3_), and ferric chloride (FeCl_3_), into the hydrogel networks to impart excellent ionic conductivity up to 10 S m^−1^.^[^ ^46^, ^47^, ^48^, ^49^ ^]^ Such high ionic conductivity is facilitated by both ion solvation and the formation of ion conduction channels within the hydrogel network. In addition, the dehydration issue can be mitigated by the incorporation of hygroscopic salts, which can reduce the vapor pressure of water by forming hydrated ions with water molecules.^[^ ^47^, ^50^ ^]^ The hydration between ions and water molecules also disrupts the hydrogen bonding network formed within water molecules, thus restricting the formation of ice and rendering the hydrogel with excellent anti‐freezing properties. Another strategy is utilizing polyelectrolytes, which carry charged groups along the chains and dissociate ions in water, as the matrix. For instance, Zhao et al.^[^ ^32^ ^]^ fabricated a sodium polyacrylate (PAANa) hydrogel fiber with high tensile strength (5.6 MPa) and stretchability (1200%), high ionic conductivity (2 S m^−1^), and great anti‐freezing property.

#### 2.1.2 Organogel‐Based Ionic Conductors

Since organic solvents have a lower freezing point than water and are relatively low in volatility, organic solvents (e.g., ethylene glycol, glycerol, dimethyl formamide, and dimethylsulfoxide) have been employed as a substitute for water in the gel systems to address the challenges of dehydration and freezing of water within hydrogels. The incorporation of organic solvents into the polymeric network leads to the emergence of organogels. By partially replacing water molecules with glycerol, glycol, and sorbitol, the organogel exhibited extreme anti‐freezing properties and resistance to drying, along with long‐term stability.^[^ ^51^ ^]^ This novel type of organogels maintained their unfrozen state and mechanical flexibility even at ‐70 °C and remained stable even in a vacuum environment. Solvent replacement is the commonly used strategy to achieve the conversion of hydrogel to organogel. For example, Song et al.^[^ ^52^ ^]^ wet‐spun the hydrogel fibers by injecting the polymer solution consisting of alginate, an end‐functionalized poly(ethylene glycol) (PEG) and (poly(ethylene glycol) diacrylate (PEGDA) into calcium chloride (CaCl_2_) solution, and subsequently covert them into organogel fibers through the replacement of water to glycerol. The resulting organogel fibers exhibited outstanding anti‐freezing capabilities (down to < ‐80 °C), remarkable stability (lasting > 5 months), high transparency, and good stretchability.

#### 2.1.3 Ionogel‐Based Ionic Conductors

Ionogels consist of polymer networks and ionic liquids. Ionic liquids, which are molten salts composed of cations and anions, remain in a liquid state at room temperature and possess a wide electrochemical window and negligible vapor pressure.^[^ ^53^ ^]^ In comparison with hydrogels and organogels, ionogels exhibit superior stability at high temperatures (up to 200 °C), attributed to the high thermal stability of ionic liquids. The properties of iongels are highly associated with the selection of ionic liquid solvents (e.g., 1‐ethyl‐3‐methylimidazolium dicyanamide ([EMIM][DCA]), 1,2‐dimethyl‐3‐ethoxyethyl‐imidazoliumbis(trifluoromethanesulfonyl)imide ([DEIM][TFSI]), N‐butyl pyridinium tetrafluoroborate ([BPy][BF_4_])), which can significantly regulate the mechanical strength, ionic conductivity, and hydrophilicity of the gels. For example, Wang et al.^[^ ^54^ ^]^ fabricated ultra‐tough (fracture strength ≈12.6 MPa) and stretchable (fracture strain ≈600%) ionogels by copolymerizing acrylamide (AM) and acrylic acid (AA) with distinct solubility of the corresponding polymers in 1‐ethyl‐3‐methylimidazolium ethyl sulfate (EMIES), which resulted in a phase‐separated network. The combination of polyvinyl alcohol (PVA) and polyacrylic acid (PAA) as polymer, and [EMIM][DCA] as ionic liquid was used to improve the ionic conductivity of ionogels up to 3.18 S m^−1^.^[^ ^36^ ^]^ Hydrophobic bis(trifluoromethanesulfonyl)imide ([TFSI]^−^) anion was further used to develop ionotronic devices underwater.^[^ ^55^ ^]^ While ionic liquids and ionogels exhibit excellent deformability, their conductivity remains orders of magnitude lower than that of electronic conductors. To address this limitation, liquid metal (LM) was incorporated into an ionogel matrix to develop a hybrid transparent electrode that combines high stretchability with superior electrical performance. This approach enables the fabrication of highly deformable matrix displays with uniform emission intensity. The integration of LM dramatically reduces the sheet resistance from 5.3 kΩ sq^−1^ to an ultralow 1.2 Ω sq^−1^, while maintaining an 81.6% optical transmittance across the entire electrode area.^[^ ^56^ ^]^ While the use of ionic liquids to solvate gels offers greater flexibility in regulating properties, it is noted that ionic liquids are more costly and viscous compared to water.

### 2.2 Solid‐State Ionic Conductors

Distinct from gel‐state ionic conductors, solid‐state ionic conductors, namely ionoelastomers, do not incorporate solvents yet still exhibit excellent stretchability (≈1000%) and good ionic conductivity (10^−5^–10^−2^ S m^−1^).^[^ ^57^ ^]^ Ionoelastomers can be formed in two ways. One method involves dissolving a polymer matrix and ionic salts in a solvent. After that, the solvent is evaporated to obtain the liquid‐free ionoelastomers. Another approach is to either mix monomers and salts to obtain a polymerizable liquid or graft charged groups onto the monomer, and subsequently polymerize the resulting mixture to form ionoelastomers. The inherent stretchability of ionoelastomers stems from the highly flexible molecular chains and the weak, reversible intermolecular forces without the plasticization of solvents. The principal advantage of ionoelastomers lies in their outstanding stability, as they are free from solvent leakage during deformation. Moreover, the ionoelastomers exhibit excellent electrochemical stability. When the applied voltage exceeds 1 V, the current flowing through the ionoelastomer remains stable across the entire voltage range without water decomposition, which is superior to that of the hydrogel where the current shows a sharp increase accompanied by the decomposition of water.^[^ ^58^ ^]^ Depending on the diverse types of components, ionoelastomers can be categorized into elastomers with organic salts, poly(ionic liquids), and poly(deep eutectic solvents).

#### 2.2.1 Elastomers with Organic Salts

Elastomers with organic salts are one type of solid‐state ionic conductors that are fabricated by dissolving organic salts into liquid monomers followed by polymerization. The monomers include ethers, esters, and siloxanes, such as oligoethylene glycol methyl ether acrylate (OEGA), ethylene glycol methyl ether acrylate (MEA), butyl acrylate (BA) and isobornyl acrylate (IBA). Organic lithium salts‐based ionoelastomers have been widely investigated, with the ionic conduction of Li^+^ facilitated by the hopping or chain segmental motion of polymer chains.^[^ ^39^ ^]^ Molecular chain engineering strategies have been adopted to tune the mechanical strength and ionic conductivity by incorporating ethylene oxide groups into the side chain. For example, Shi et al.^[^ ^59^ ^]^ presented an ionic‐conducting elastomer composed of poly(butyl acrylate) (PBA), polyethyleneglycol diacrylate (PEGDA), and lithium bis(trifluoromethane sulfonimide) (LiTFSI), which exhibited high‐temperature stability, high‐voltage stability, and non‐corrosiveness towards metal electrodes. The ionic conductivity of the ionoelastomer was measured at 1.27 × 10^−5^ S m^−1^ at 20 °C. To achieve higher ionic conductivity, Shi et al.^[^ ^40^ ^]^ developed a liquid‐free ionic elastomer (ionic conductivity ≈3.5 × 10^−3^ S m^−1^) featuring microphase‐separated structures, where the hydrophilic nanodomains offered highly efficient conductive pathways for Li⁺ ions. In addition to side‐chain design, main‐chain engineering for solid‐state ionic conductors has also been studied. Mackanic et al.^[^ ^60^ ^]^ reported on supramolecular Li^+^ ion conductors that employed orthogonally functional hydrogen‐bonding domains and ion‐conducting domains, leading to the creation of a polymer electrolyte exhibiting extraordinary toughness (29.3 MJ m^−3^) and high ionic conductivity (1.2 × 10^−2^ S m^−1^ at 25 °C).

#### 2.2.2 Poly(Ionic Liquids)

Poly(ionic liquids) (PILs)‐based ionoelastomers represent another category of liquid‐free ionic conductors, which are the polymerization products of ionic liquid monomers. The distinction between ionogels and PILs lies in the fact that PILs are liquid‐free. Unlike functioning as solvents and conducting ions in ionogels, ionic liquids serve as the monomers in PILs and are subsequently polymerized to form a polymer network with mobile cations or anions. Typically used polymerizable ionic liquid monomers are 1‐vinyl‐3‐methylimidazolium chloride ([VMIM]Cl), 1‐vinyl‐3‐ethylimidazolium bis(trifluoromethanesulfonyl)amide ([VEIM][TFSA]), and 1‐vinyl‐4‐methylpyridinium bromide ([VMPy]Br). Owing to the presence of ionic liquids species within their repeating unit, poly(ionic liquids) can display tunable physical and chemical properties, including solubility, ion conductivity (10^−4^–10^−1^ S m^−1^), electrochemical stability (up to 5 V versus Li^+^/Li^0^), thermal stability (up to 300 °C), and nonflammability.^[^ ^61^ ^]^ The specific manifestation of these properties depends on the type of cation‐anion pair, a characteristic analogous to that of ionic liquids. For solubility, cations with long alkyl chains lead to reduced solubility in polar solvents. Ionic conductivity is significantly influenced by the cation‐anion pair. Cations with low charge density and high mobility, such as alkylammonium cations, can facilitate the movement of ions within the PILs matrix. The choice of anion also matters; anions that can dissociate easily and have good mobility, like [TFSI]^−^, can enhance the overall ion conductivity. The strong ion‐ion interactions between cation‐anion pairs may limit the mobility of ions and thus reduce the conductivity but endow better thermal stability.^[^ ^61^ ^]^Nevertheless, similar to ionogel, the use of costly ionic liquids restricts the widespread production of PILs‐based ionoelastomers.

#### 2.2.3 Poly(Deep Eutectic Solvents)

To reduce the production cost of ionic conductors, polymerizable deep eutectic solvents (DESs) systems have emerged as an alternative to ionic liquids. DESs, which are low‐melting eutectic liquids composed of a mixture of hydrogen bond donors (HBDs) and acceptors (HBAs), exhibit low volatility, high thermal stability, good ionic conductivity, and low cost.^[^ ^62^ ^]^ Monomers containing hydroxyl, carboxyl, or amide groups can function as hydrogen bond donors, while salts act as hydrogen bond acceptors. By simply heating these components for a certain period, DESs are formed, and subsequent polymerization yields liquid‐free ionic conductors (i.e., Poly(deep eutectic solvents) (PDESs)). For instance, Fang et al.^[^ ^43^ ^]^ developed tough (toughness ≈38 MJ m^−3^) and ionic conductive (6 × 10^−3^ S m^−1^) PAA/ChCl/LiCl PDESs fibers through one‐pot photopolymerization and lithium cation‐induced toughening effects. Another novel liquid‐free PDESs was developed through radical polymerization of acrylic acid (AA) with choline chloride (ChCl) and liquid metal (LM). Ultrasonic processing of the LM/AA mixture generated nanoscale droplets that served dual functions: initiating the free‐radical polymerization of poly(diethyl siloxane) while simultaneously releasing Ga^3+^ cations to crosslink the resulting poly(acrylic acid) (PAA) networks. This unique synthesis strategy yielded a eutectogel exhibiting exceptional optical transparency (94.1% transmittance) and remarkable stretchability (2600% strain), enabled by the dynamic coordination bonds between Ga^3+^ and carboxylate groups in the polymer matrix.^[^ ^63^ ^]^ However, PDESs tend to absorb water from the atmosphere due to the hygroscopic nature of their components. To ensure the solid state of PDESs, hydrophobic components, such as organic compounds with long alkyl chains, can be selected for their preparation. For example, Du et al.^[^ ^44^ ^]^ prepared hydrophobic DES by mixing non‐hygroscopic tetrabutylammonium tetrafluoroborate (N_4444_BF_4_) and amphiphilic trifluoroacetamide (TFA). Then the hydrophobic PDESs were constructed by copolymerizing 2,2,3,4,4,4‐hexafluorobutyl acrylate (HFBA) and isobornyl acrylate (IBOA) in DES for underwater sensing and adhesion.

### 2.3 Shaping Ionic Conductors into Textiles

1D fibers and 2D fabrics and textiles, with their distinct structural features, have emerged as promising formats for wearable electronics/ionotronics. The following sections summarize the advantages of shaping ionic conductors into 1D fibers and 2D fabrics. Specially, the fabrication methods will be discussed in Section 4.

#### 2.3.1 1D Fiber‐Shaped Ionic Conductors

One of the prominent advantages of 1D ionic conductive fibers lies in their high aspect ratio, which leads to a substantial surface‐area‐to‐volume ratio. In fiber‐shaped chemical sensors, for example, the large surface area facilitates more efficient interactions between substrate and target analytes, exhibiting faster response times and enhanced sensitivity compared to bulky film‐shaped sensors. Moreover, the flexibility of fibers renders ionic conductors suitable for applications where conformability is of importance. In wearable displays, these fibers can be employed as flexible and stable electrodes, which can endure bending and twisting without significant degradation in mechanical performance and ionic conductivity, thereby enabling the fabrication of displays that can be rolled or folded. The 1D fibers can further be seamlessly integrated into intricate 2D fabrics and 3D structures.

#### 2.3.2 2D Fabric‐Shaped Ionic Conductors

2D fabrics composed of ionic conductors possess a planar surface area, which is highly beneficial in applications such as fuel cells. In a textile‐based fuel cell, the extensive surface area of the fabric can accommodate a greater number of catalyst sites, thereby leading to a higher power output by enhancing the electrochemical reactions involved in fuel cell operation. Besides, 2D fabric‐shaped ionic conductors can be integrated into clothing to create smart garments. For example, a textile‐based electrocardiogram (ECG) sensor made of ionic conductive fabric can adhere comfortably to the skin and accurately detect electrical signals from the human body.^[^ ^64^ ^]^ Users can move freely while wearing the device due to the flexibility and breathability of fabrics, in contrast to rigid or bulky health monitoring equipment.

## 3 Properties of Textile‐Based Ionic Conductors

Shaping ionic conductors into fibers and other textile formats is emerging as a pivotal element in the development of wearable technology and E‐textiles, owing to their remarkable blend of ionic conductivity, mechanical flexibility, and feasibility of integration into wearable formats. For effective integration into wearable devices, these conductors must balance functional performance with user comfort. Essential properties that textile‐based ionic conductors need to be considered may include: i) mechanical properties that can endure repeated stretching and bending without failure, which are crucial for extending the lifespan of the devices and reducing the need for frequent replacements and minimizing waste; ii) efficient ionic conduction to facilitate rapid signal transmission, which is highly essential to the functionality of ionotronics; iii) biocompatibility to ensure that textile‐based ionic conductors do not induce harmful reactions upon skin contact; iv) sustainability to reduce the resource depletion and waste accumulation through recycling and biodegrading design.

### 3.1 Mechanical Properties

**Figure 3:**
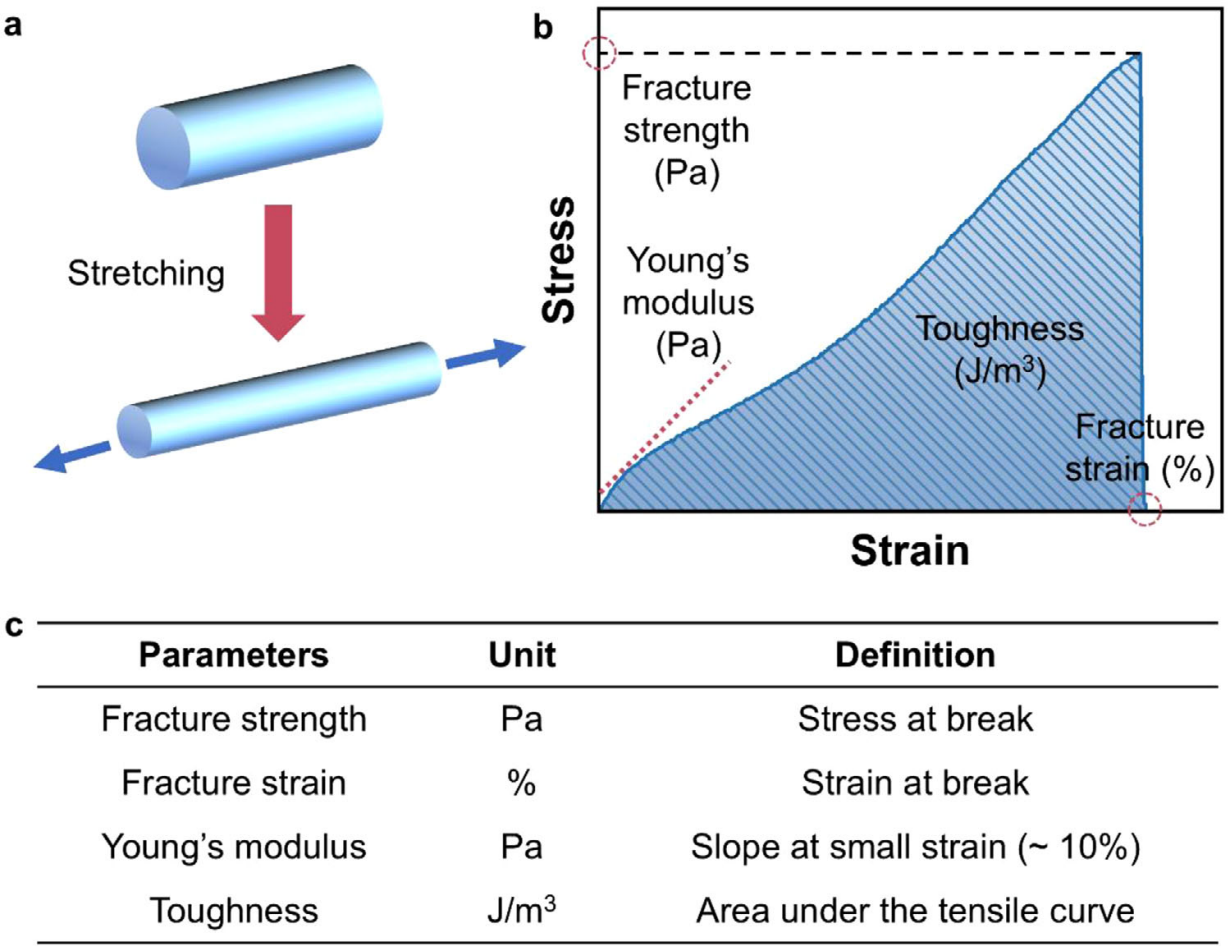
a) The tensile measurement of ionic conductors. b) Stress–strain curves of ionic conductors. c) The definition of mechanical parameters of ionic conductor fibers.

The application of textile‐based ionic conductors is largely influenced by their mechanical properties, which are reflected in their ability to withstand high stress and large strain, as well as their structural stability upon cyclic deformation (**Figure** **3**a). Key parameters that are commonly assessed to evaluate the mechanical properties of these fibrous materials include fracture strength, fracture strain, Young's modulus, and toughness. They can be derived from stress‐strain curves (Figure 3b).

Fracture strength is the maximum tensile stress that a material can withstand before failure or fracture occurs (Figure 3c). High fracture strength of ionic conductive fibers, especially exceeding 10 MPa, is particularly beneficial for the machine manufacturing of textile‐based ionic conductors, as it enables the fibers to withstand the mechanical stress exerted by needles or hooks and maintain structural integrity under friction and cyclic deformation.^[^ ^65^, ^66^ ^]^ Otherwise, insufficient breaking strength can result in frequent yarn breakage because of the high tension of the loom during manufacturing (e.g., warp tension in weaving or hook pulling in knitting). For weaving, industrial weaving looms exert warp tensions up to 500 MPa (e.g., polyester benchmark yarns), necessitating high‐strength fibers to prevent breakage during shedding and beat‐up.^[^ ^67^ ^]^ In knitting, fibers undergo cyclic bending and tensile loading during loop formation. A fracture strength lower than 10 MPa leads to failure due to hook‐induced deformation (e.g., a 4.5 MPa fiber fractured during needle penetration, whereas a 12 MPa variant exhibited stable loop formation).^[^ ^65^ ^]^ Fracture strain, usually represented as a percentage or a ratio, indicates the change in the length of the material relative to its original length before the application of stress, providing valuable insight into the ductility and deformability of a material. A larger fracture strain indicates that a material can undergo substantial deformation prior to breaking. The high deformability is advantageous in applications of wearable electronics and flexible displays, where both flexibility and resilience are essential requirements.

**Figure 4:**
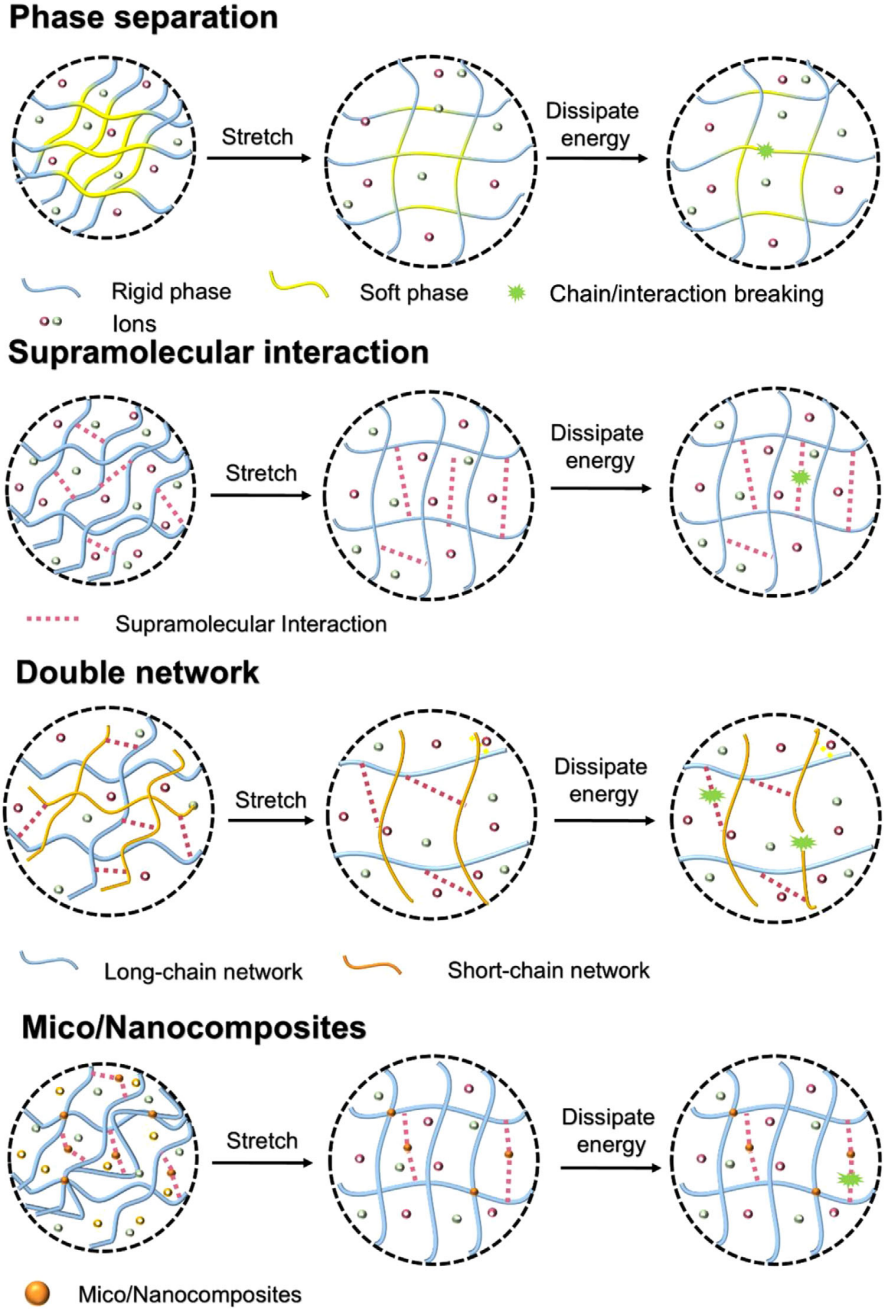
Schematics illustrating the toughening strategies in ionic conductors, including phase separation, supramolecular interaction, double network, and micro/nanocomposites.

Young's modulus, which quantifies the stiffness of a material, is calculated from the slope of the stress‐strain curve in the elastic region (at low strain ≈10%), where the material exhibits linear elastic behavior. A high Young's modulus indicates that a material is stiff and requires a large amount of stress to produce a small strain, while a low Young's modulus suggests greater material flexibility, enabling it to deform more readily under applied stress. Toughness, representing the energy dissipated per unit volume before fracture, is determined by integrating the area under the stress‐strain curve up to the point of failure. In applications prone to sudden impacts or requiring materials to endure substantial deformation without fracture, such as in construction, automotive, and aerospace industries, materials with high toughness are highly sought after. Despite their potential, many fiber‐based ionic conductors encounter significant mechanical challenges, such as insufficient strength and limited toughness for machine weaving, susceptibility to crack propagation, and a tendency to experience fatigue damage. To address these issues and enhance the mechanical properties of fiber‐based ionic conductors, it is essential to rationally design their structures and incorporate effective energy dissipation mechanisms. Recent advancements have introduced various strategies for strengthening these materials, including the implementation of phase separation techniques,^[^ ^54^, ^68^, ^69^ ^]^ the incorporation of supramolecular interactions,^[^ ^70^, ^71^ ^]^ the development of double networks,^[^ ^72^, ^73^ ^]^ and the use of micro/nanocomposites (**Figure** **4**).^[^ ^74^ ^]^ Collectively, these approaches have resulted in substantial improvements in the mechanical performance of textile‐based ionic conductors, thereby enhancing their reliability and applicability in various fields.

#### 3.1.1 Phase Separation

To enhance the properties of textile‐based ionic conductors, specifically their stiffness and toughness, a rigid‐soft microphase separation structure can be designed. The rigid phase functions as a high‐energy barrier that minimizes network mobility, thereby improving the stiffness of the polymer network. In contrast, the soft phase allows for extensive deformation, which dissipates energy and enhances toughness. This combination of rigid and soft phases results in a polymer network that exhibits improved performance in both stiffness and toughness. For example, Zhang et al.^[^ ^75^ ^]^ developed a fiber‐based ionic conductor featuring a rigid‐soft microphase‐separated polymer network, which exhibited unprecedented mechanical properties. Poly(vinyl alcohol) (PVA), known for its ability to form hydrogen bonds and create rigid crystalline domains, was selected as the rigid phase. The PDES, obtained through the polymerization of choline chloride and acrylic acid, was interwoven with amorphous PVA chains to establish the soft phase. This meticulously designed microphase‐separated structure endows the ionic conductor with excellent mechanical properties, including enhanced stiffness (128.6 MPa), toughness (134.7 MJ m^−3^), remarkable mechanical strength (37.5 MPa), and improved resistance to tearing and crack propagation.

#### 3.1.2 Supramolecular Interaction

Supramolecular interactions such as hydrophobic association ionic bonds, hydrogen bonds, and electrostatic interactions, can be incorporated into polymer networks as sacrificial bonds to improve the mechanical properties. Notably, these noncovalent bonds possess inherent reversibility, which allows fiber‐based ionic conductors to exhibit a range of multifunctional capabilities, including self‐healing, recovery, and shape memory. For example, Song et al.^[^ ^52^ ^]^ introduced both ion‐crosslinking and covalent crosslinking in the polymer network of organohydrogel fibers. They utilized polyethylene glycol diacrylate and 2‐hydroxyethyl acrylate for covalent crosslinking, while alginate and Ca^2+^ formed an ion‐crosslinked nascent fiber capable of withstanding drafting forces before the covalent network formation. The resulting organohydrogel fibers exhibited robust elasticity, achieving a maximum elongation of 400% and negligible hysteresis during cyclic loading and unloading up to 300% strain, with minimal drift over 1000 stretch‐release cycles. Li et al.^[^ ^76^ ^]^ reported a kind of ionic polyimide hydrogel fibers that were derived from a polyimide (PI) salt solution and produced on a large scale through a continuous wet spinning process, utilizing an aqueous calcium chloride solution as the coagulating bath. During the spinning of PI hydrogel fibers, the coagulation bath played a crucial role in influencing the sol‐gel transition of the PI salt. It regulated both the gelation time and the extent of cross‐linking, which were critical factors affecting the mechanical properties and microstructures of the resulting hydrogel fibers. Calcium ions typically act as ionic complexation cross‐linkers, and solutions containing Ca^2+^ are commonly employed as coagulants in hydrogel preparation. The hydrogel fibers exhibited a maximum tensile strength of approximately 2.5 MPa and an elongation at a break of 215%, significantly surpassing the performance of fibers, which have a tensile strength of 0.65 MPa and an elongation of 77%. This enhancement in mechanical properties could be attributed to the ionic cross‐linking facilitated by Ca^2+^ ions between the polymer chains.

#### 3.1.3 Double Network

Covalent single polymer networks tend to be rigid and lack the ability to dissipate energy effectively during stretching. The incorporation of a secondary network within a single polymer matrix could improve the mechanical properties. Double‐network ionic conductors consist of two interpenetrating polymer networks that are bound through noncovalent interactions. When subjected to deformation under stress, the short‐chain network, which facilitates mechanical dissipation, will break first, thereby dissipating a significant amount of mechanical energy. Meanwhile, the long‐chain network, characterized by its high tensile strength, can undergo stretching at the molecular level while preserving the overall integrity of the polymer network, ultimately leading to enhanced toughness of the fibers. For example, Zhao et al.^[^ ^77^ ^]^ utilized modified bamboo fiber (BF), sodium alginate (SA), and sodium chloride (NaCl) as the primary raw materials to prepare hydrogel. The SA/BF composite hydrogel was produced through a rapid polymerization reaction involving a calcium chloride solution within a mold. Furthermore, the conductivity of the SA/BF composite hydrogel was enhanced by immersing it in a NaCl salt solution. The incorporation of modified BF facilitated the formation of a double network structure through hydrogen bonding, which interacted with the initial network established by the cross‐linking of Ca^2+^ ions in the sodium alginate hydrogels. This addition significantly increased the overall rigidity of the hydrogel. In comparison to a single network, the nodes in the double network hydrogel are more densely packed, resulting in improved toughness and tensile strength of the material. Experimental results indicated that with a modified bamboo fiber content of 1.5%, the mechanical properties of the hydrogel were notably enhanced, achieving an elongation at a break of 340% and a tensile strength of 1.58 MPa.

#### 3.1.4 Micro/Nanocomposites

The incorporation of nanocomposites represents an additional strategy for improving the mechanical properties of fiber‐based ionic conductors. These nanocomposites enhance the entanglement of polymer networks through interactions between the nanomaterials and the polymer chains. Furthermore, they serve as centers for stress transfer and dissipation, effectively alleviating stress concentration at the crack tip. For instance, Li et al.^[^ ^78^ ^]^ introduced a synergistic approach that utilized antisolvent doping to fabricate ionogel fibers doped with silicon dioxide (SiO_2_). The incorporation of SiO_2_ nanoparticles promoted the development of an organic‐inorganic hybrid network within the ionogel fibers, enhancing their mechanical properties. This improvement was attributed to the hydrogen bonds formed between the inorganic SiO_2_ and the polymer molecular chains. Following the addition of SiO_2_, the ionogel fibers demonstrated a tensile strength of 327 kPa and a breaking strain of 47%.

### 3.2 Ionic Conduction

The conductivity of fiber‐based ionic conductors arises from the movement of ions within their network structure. Ionic conductivity is quantitatively assessed via electrochemical impedance spectroscopy (EIS) using the electrochemical workstation. The ionic conductor is sandwiched between ion‐blocking electrodes (e.g., stainless steel, platinum (Pt), gold (Au), or silver (Ag) foil), and a sinusoidal alternating current (AC voltage is applied across a defined frequency range (from 10^−2^ to 10^5^ Hz) to record impedance spectra. The frequency‐dependent response is analyzed using Bode and Nyquist plots, alongside equivalent circuit modeling. The bulk resistance (*R*) is derived from the intersection point between the high‐frequency semicircle and the X‐axis in the Nyquist plot, enabling the calculation of ionic conductivity (*σ*) through the relation *σ = L/(RS)*, where *L* and *S* denote the thickness and cross‐sectional area of the ionic conductor, respectively.^[^ ^79^ ^]^

Therefore, the ionic conductivity of ionic conductors is influenced not only by the mobility and density of ions in the polymer network, but also by the structural characteristics of the fibers themselves. Ionic conductive textiles with high ionic conductivity are critical for signal transmission within ionotronics, allowing the accurate detection of physiological signals like electrocardiogram (ECG) and electromyogram (EMG) signals for healthcare management.

**Figure 5:**
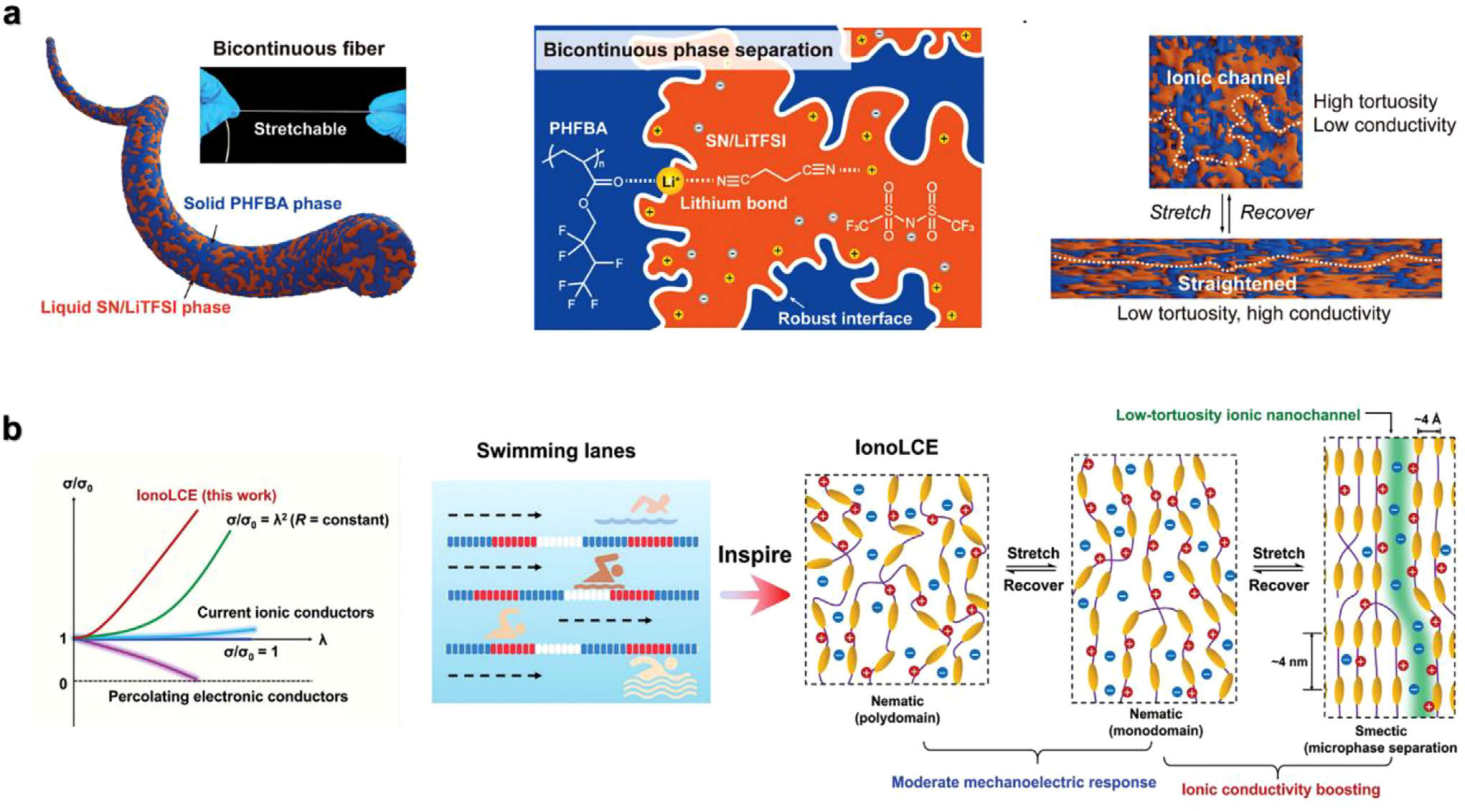
a) Schematic illustration and a photo of the stretchable bicontinuous fiber. Schematic structure of the fiber with polymerization‐induced bicontinuous phase separation. Working mechanism for strain‐insensitive ionic conduction. Reproduced with permission.^[^ ^80^ ^]^ Copyright 2024, Wiley‐VCH. b) Mechanoelectric response of IonoLCE fiber in comparison with common electronic and ionic conductors. Swimming lane‐inspired working mechanism of IonoLCE. Reproduced with permission.^[^ ^81^ ^]^ Copyright 2021, Wiley‐VCH.

Ion transport channel creation is the key to ensuring ion conduction and thereby determining the ionic conductivity of materials. A microphase‐separated structure, which results from solid‐liquid mixtures and is characterized by two interlinked and continuous phases, can be considered as a way to create ion transport channels. This phenomenon facilitates the formation of a porous structure that allows conductive ions to transport within a liquid phase, thereby imparting novel functionalities to various natural and synthetic materials. The microphase‐separated structure has shown considerable promise in mitigating the strain‐induced increase in resistance. When stretched, the ionic pathway transitions from tortuous to linear, increasing the ionic conductivity via reduced ion transport barriers. This counterbalances the strain‐induced pathway elongation, resulting in a slight resistance change under stretching. For example, Ye et al.^[^ ^80^ ^]^ synthesized a fiber with a solid‐liquid bicontinuous microstructure by polymerizing hexafluorobutyl acrylate (HFBA) in the presence of succinonitrile (SN) and lithium bis(trifluoromethanesulfonyl)imide (LiTFSI). During polymerization, phase separation occurred, resulting in a fiber structure with a solid elastomer phase interpenetrated by a liquid ion‐conducting phase (**Figure** **5**a). LiTFSI is distributed into the SN phase, creating interconnected ion‐rich liquid domains. Additionally, the ion‐dipole interactions between Li⁺ ions and carbonyl groups (lithium bonds) led to a strong self‐rinkled interface at the phase boundary. This bicontinuous architecture gave the fiber excellent properties, including 750% stretchability, 0.04 S m^−1^ ionic conductivity, and outstanding anti‐fatigue performance. Even when strained by 200%, the fiber maintained highly stable ionic conduction with only a 7% resistance increase. Yao et al.^[^ ^81^ ^]^ reported an ionotronic fiber composed of ionic liquid and liquid crystal elastomer, which exhibited unprecedented strain‐induced ionic conductivity. These alternating rigid mesogen units and soft chain spacers of ionotronic fibers formed a microphase‐separated structure with low‐tortuosity ion‐conducting nanochannels. Aligned smectic mesophases induced by strain‐guided ultrafast ion transport, similar to “swimming channels”. When stretched to 2000%, the fiber enhanced its ionic conductivity by a factor of 1000 (Figure 5b).

Ionic conductors primarily derive their conductivity from two main sources: zwitterionic polymers and free ions. Free ions are favored over zwitterionic polymers due to their flexibility and unique characteristics. However, the polymer chain would impede the movement of ions, presenting a trade‐off between conductivity and mechanical strength. Developing efficient ion transport channels can address this issue. For example, Deng et al.^[^ ^82^ ^]^ transformed natural cotton into ionic‐electronic conductive fibers. The cellulose component of cotton provides a multiscale interspace and aligned nanochannel scaffold for effective ion transportation. The hydrophilic cellulose wall also promoted the infiltration of liquid ink like PEDOT:PSS, forming robust composite fibers. These fibers showed high ionic and electronic conductivities of 2 S m^−1^ and 5 S m^−1^, respectively, even under 175% strain.

### 3.3 Biocompatibility

When textile‐based ionic conductors are utilized in wearable technology, direct contract with the human body is essential. Consequently, biocompatibility emerges as a critical property of these materials. Biocompatibility minimizes the risk of adverse reactions, such as irritation, inflammation, or allergic responses, thereby ensuring that the ionic conductors can interact safely and effectively with biological tissues. This characteristic is vital for enhancing user comfort and safety, ultimately facilitating the successful application of wearable devices in health monitoring and other biomedical contexts.

To achieve excellent biocompatibility in textile‐based ionic conductors, the selection of biocompatible polymers as matrix and non‐toxic salts as conductive ions, as well as green solvents, is important. Among the common choices are natural polymers (such as chitosan, alginate, or cellulose) and synthetic polymers (for example, polycaprolactone (PCL) or polylactic acid (PLA)) that have demonstrated biocompatibility. For example, Chen et al.^[^ ^83^ ^]^ utilized biocompatible sodium alginate as the base material and non‐toxic inorganic salt CaCl_2_ as the conductive component to fabricate biocompatible hydrogel ionotronic fibers through a wet‐spinning process. To assess the biocompatibility of these fibers, bone mesenchymal stem cells (BMSCs), a commonly used adult stem cell line, were cultured in extracts derived from the fabricated fibers. After 1, 3, and 5 days of cell culture, the cell viability on the alginate hydrogel ionotronic fibers (AHIFs) was found to be comparable to that of conventional culture medium. Furthermore, when BMSCs were seeded onto the surfaces of the AHIFs, their growth was similar to that observed when cultured directly on polystyrene cell culture plates. These results demonstrated the excellent biocompatibility of AHIFs in promoting cell growth, highlighting their potential for applications in tissue engineering and regenerative medicine. In another study, Cheng et al.^[^ ^84^ ^]^ fabricated choline chloride and mannose‐based deep eutectic solvents (ChCl/M DES), followed by the introduction of lysozyme fibers and gallic acid to create injectable eutectogels through self‐assembly via hydrogen bonding and hydrophilic/hydrophobic interactions. The cytotoxicity of these eutectogels was evaluated on human gingival fibroblasts (HGF) and human oral keratinocytes (HOK) over a 7‐day period using a cell counting kit‐8 (CCK‐8) assay, live/dead staining, and morphological staining. The measured cell viability showed no significant difference from the control group across all experimental groups. These findings further underscore the importance of biocompatibility in the development of fiber‐based ionic conductors for biomedical applications.

### 3.4 Sustainability

In the field of wearable electronics, the use of some toxic materials (e.g., fluorocarbons and highly reactive monomers) is critical in industries ranging from textiles (e.g., finishing and post‐treatment) to electronics (e.g., semiconductor fabrication). However, their persistence, bioaccumulation potential, and toxicity raise significant environmental concerns. Synergizing recyclable and biodegradable designs will be pivotal for developing sustainable ionotronic devices that align with circular economy principles, minimizing resource depletion while maintaining high performance.

Recyclability stands as a cornerstone for advancing the sustainability of next‐generation electronics, transforming end‐of‐life devices into reusable resources while mitigating environmental burdens. An ideal recycling paradigm must satisfy four critical criteria, including economic viability to ensure cost‐competitiveness with virgin material production, operational simplicity to enable scalable implementation, high‐value material recycling to maximize resource efficiency, and minimal energy consumption to reduce carbon footprints. Notably, the economic and ecological advantages of recycling become compelling when the process costs are substantially lower than those of conventional fabrication, which is a key consideration for ionically conductive polymers and fibers. For fiber‐based ionic conductors, recycling can be implemented across multiple strategies: i) mechanical recycling via shredding and reprocessing to preserve ionic networks;^[^ ^85^ ^]^ ii) polymer reprocessing through melting/dissolution to regenerate functional fibers;^[^ ^86^, ^87^ ^]^ and iii) chemical recycling by depolymerizing into monomers for closed‐loop synthesis, particularly critical for petroleum‐derived systems.^[^ ^88^ ^]^

Biodegradability further complements recyclability by ensuring environmentally benign degradation when recycling is impractical. Biodegradable ionic conductors decompose into non‐toxic byproducts via oxidation, photolysis, or microbial action, circumventing persistent waste accumulation.^[^ ^89^ ^]^ Biodegradable ionic conductors are classified by origin: i) biomass‐derived systems (e.g., cellulose, chitosan, protein), which leverage renewable feedstocks to form intrinsically biodegradable ionic conductive matrices with minimal ecological impact;^[^ ^90^, ^91^ ^]^ and ii) engineered petroleum‐based systems, where synthetic polymers (e.g., polycaprolactone) incorporate hydrolyzable/oxidable linkages to achieve controlled degradation despite their fossil‐fuel origins.^[^ ^92^ ^]^

## 4 Fabrication and Integration Technologies of Ionic Conductive Textiles

Ionic conductive fibers are the fundamental building blocks for constructing flexible and wearable ionic textiles. Ionic conductive 1D fibers possess an elongated morphology, enabling the efficient transport of ions along the axial direction of the fibers. As for ionic conductive 2D fabrics or textiles, they are formed by interweaving or knitting fibers, which have a large specific surface area, and ions can conduct within the network structure formed by the interwoven fibers. Meanwhile, 2D fabrics or textiles have certain flexibility and stretchability, making them suitable for fabricating large‐area, body‐conforming ionic devices. Various fabrication methods have been developed to create fiber‐based ionic devices at the single‐fiber level and within fabrics/textiles. These methods can be categorized into two types: the spinning of ionic conductive fibers and the manufacturing of ionic conductive textiles.

### 4.1 Spinning of Ionic Conductive Fibers

Ionic conductive fibers serve as the fundamental building blocks for smart fabrics and textiles. In this section, the fabrication methods of ionic conductive fibers with diameters spanning from nanometer to millimeter scales are discussed in detail, including wet spinning, draw spinning, thermal spinning, dynamic crosslinking spinning, and electrospinning (**Table** **2**). Moreover, the advantages and limitations of each method are also summarized, with particular emphasis on the demands of sustainable manufacturing techniques of ionic conductive fibers, such as less waste of materials, less solvent use, and less energy‐intensive processes.

**Table 2:** Summary of spinning methods of fiber‐shaped ionic conductors.

Spinning methods	Materials^a)^	Diameter [µm]	Fracture strength [MPa]	Fracture strain [%]	Young's modulus [MPa]	Toughness [MJ m^−3^]	Ionic conductivity [S m^−1^]	Refs.
Wet spinning	Gelatin‐(NH_4_)_2_SO_4_ hydrogel	900	16.4	658	0.44	4.4	5.2	[93]
	NaAlg‐CaCl_2_ hydrogel	300–1500	≈0.7	≈300	≈5	≈0.4	0.105	[83]
	SF‐[EMIM][BF_4_] ionogel	130‐450	55	530	≈80	≈12.5	0.45	[94]
Draw spinning	PrDA hydrogel	5‐50	50‐275	40‐65	120‐3000	13‐125	0.3‐6.5	[95]
	SF‐LiCl/FA organogel	200	1.4	300	≈3	≈8	0.58	[96]
	PAN‐AgNO_3_/DMF organongel	130	≈6	≈500	≈2	≈13	1.82	[97]
Thermal spinning	P(AM‐co‐NAGA)‐LiCl hydrogel	500	2.27	≈900	≈0.15	≈9	0.69	[98]
	PVA/PEI‐LiCl hydrogel	100‐800	1.7	>7000	≈8	≈0.15	3.6	[99]
Dynamic crosslinking spinning	P(AM‐co‐AA)‐FeCl_3_ hydrogel	550‐950	6.8	≈600	5.4	30.08	8.4 × 10^−2^	[100]
	PVA/PAA‐Zr/NaSO_4_/Gly organogel	900	24.43	≈900	36.81	162.25	0.1	[101]
Electrospinning	TPU‐[EMM][TFSI] nanofiber	0.61	≈17	≈180	≈14	≈18	8.57 × 10^−3^	[102]
	[PBVIm][TFSI] nanofibrous membrane	1.12	≈15	≈50	≈30	≈4	≈6.82 × 10^−3^	[103]

^a)^ NaAlg: sodium alginate, SF: silk fibroin, [EMIM][BF_4_]: 1‐ethyl‐3‐methylimidazolium tetrafluoroborate, PrDA: proton donor‐acceptor, FA: formic acid, PAN: polyacrylonitrile, DMF: dimethyl formamide, P(AM‐co‐NAGA): poly(acrylamide‐co‐N‐acryloylglycinamide), PVA: polyvinyl alcohol, PEI: polyethyleneimine, P(AM‐co‐AA): poly(acrylamide‐co‐acrylic acid), Zr: zirconium, Gly: glycerol, TPU: thermoplastic urethanes, [EMIM][TFSI]: 1‐ethyl‐3‐methylimid‐azolium dicyanamide bis(trifluoromethanesulfonyl)imide, [PBVIm][TFSI]: poly (1‐butyl‐3‐vinylimidazolium bis(trifluoromethanesulfonyl)imide).

#### 4.1.1 Wet Spinning

Wet spinning, a mature method for large‐scale fiber fabrication in the textile industry, has been widely employed in the fabrication of ionic conductive fibers. In the spinning process, the solution solidifies into a fiber‐shaped form within the coagulation bath. Based on distinct solidification mechanisms, wet spinning can be classified into three types: i) salting‐out effects induced solidification, ii) ionic crosslinking induced solidification, and iii) antisolvent exchange‐induced solidification.

**Figure 6:**
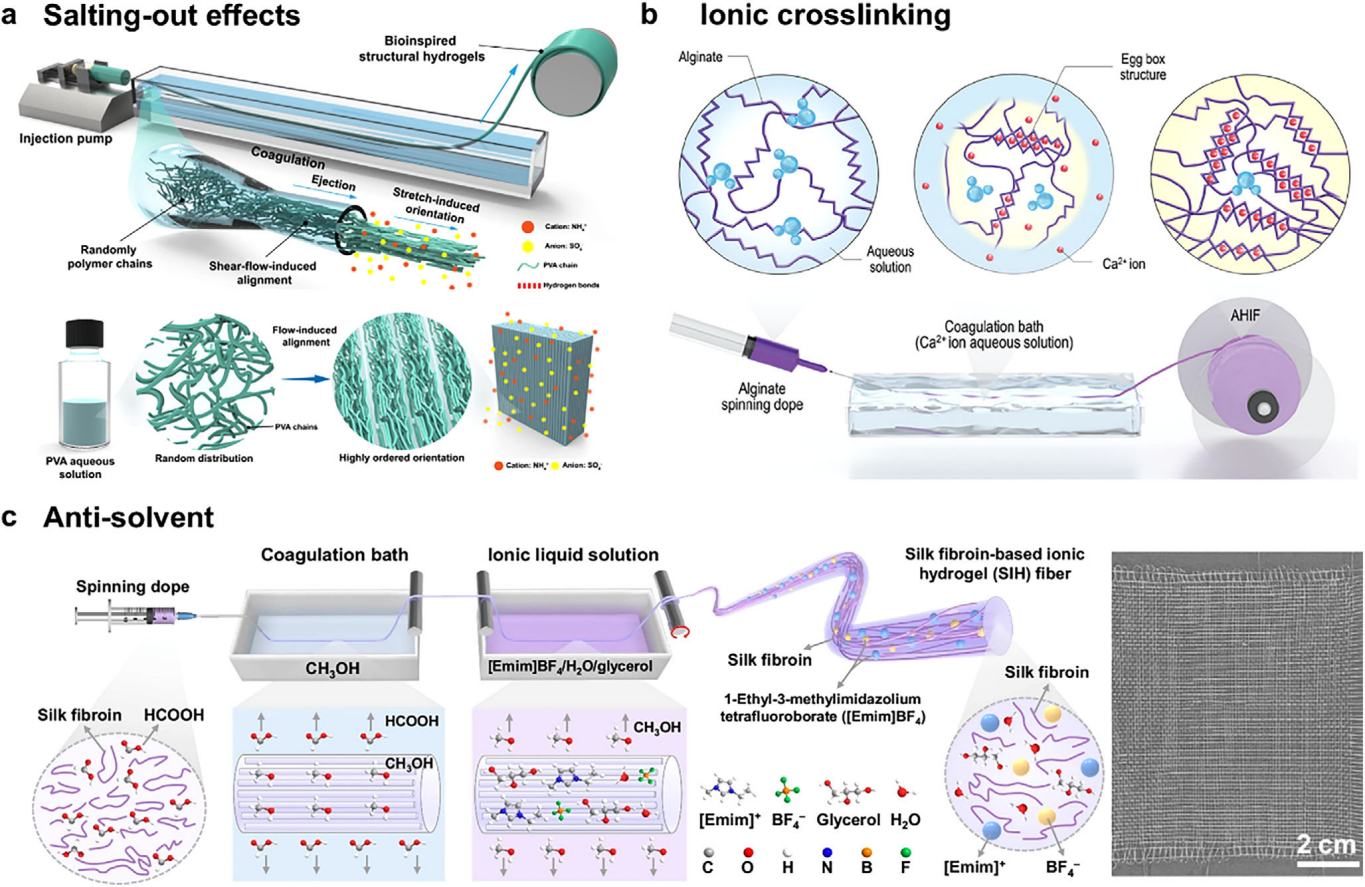
a) Fabrication procedure for well‐ordered nanofibrils using flow‐induced alignment. Randomly distributed PVA polymer chains transform into a highly oriented hydrogel network by flow‐induced alignment. Reproduced under the terms of the CC‐BY Creative Commons Attribution 4.0 International license (https://creativecommons.org/licenses/by/4.0/).^[^ ^104^ ^]^ Copyright 2024, published by Springer Nature. b) Schematic of the ionic chelation‐assisted wet‐spinning technique and the molecular structure changes of alginate during the wet‐spinning process. Reproduced with permission.^[^ ^83^ ^]^ Copyright 2022. Royal Society of Chemistry. c) Scheme diagram for the fabrication of SIH fibers and a plain weave textile made of SIH fibers demonstrating the weavability of SIH fibers. Reproduced under the terms of the CC‐BY Creative Commons Attribution 4.0 International license (https://creativecommons.org/licenses/by/4.0/).^[^ ^94^ ^]^ Copyright 2024, published by Springer Nature.

In wet spinning assisted by salting‐out effects and ionic crosslinking, the coagulation bath is composed of salt solutions. Through the diffusion of ions, the coagulation bath solidifies the fiber shape and directly endows fibers with ionic conductivity. When leveraging salting‐out effects, in the presence of kosmotropic ions (e.g., ammonium sulfate), the aggregation and crystallization of polymer chains (e.g., polyvinyl alcohol, gelatin, and sodium alginate) facilitate the coagulation of the spinning solution. Besides enabling rapid and controllable fiber solidification, the shear flow and extensional flow contribute to the highly ordered hierarchical orientations of polymer chains, which results in excellent mechanical properties and insensitivity to cracks (**Figure** **6**a).^[^ ^104^ ^]^ Apart from enhancing interaction between polymer chains in salting‐out effects, ions could also act as crosslinking sites to bind the amorphous chains.^[^ ^105^ ^]^ A typical example is the extrusion of sodium alginate spinning dope into the Ca^2+^ coagulation solution, during which the Ca^2+^ ions quickly diffuse into the dope and trigger an exchange between Ca^2+^ and Na^+^ ions (Figure 6b).^[^ ^83^ ^]^ The bivalent Ca^2+^ ions are chelated with alginate molecules to form an egg‐box‐like structure to facilitate the alginate gelation. This alginate‐Ca^2+^ hydrogel has often been selected as the shell layer to fabricate fibers with a core‐shell structure.^[^ ^106^, ^107^ ^]^

Wet‐spinning by the anti‐solvent method applies to a variety of polymer systems. Phase separation occurs during the spinning process due to the different solubilities of the polymers in solvents and antisolvents.^[^ ^94^, ^108^, ^109^ ^]^ For instance, Lu et al.^[^ ^94^ ^]^ prepared a mechanically strong, ionic conductive, and stable silk fibroin‐based hydrogel fiber by extruding fibroin/formic acid solution into a methanol coagulation bath and passing through an ionic liquid bath (Figure 6c).

In summary, wet spinning enables the continuous fabrication of ionic conductive gel fibers with exceptional uniformity and mechanical robustness, making it a promising approach for scalable production of textile‐based ionotronics. However, the reliance on coagulation baths introduces inherent limitations, including solvent waste generation and potential environmental contamination, particularly when employing costly or toxic solvents. Furthermore, the slow solidification kinetics may compromise production throughput, posing challenges for high‐speed manufacturing.

#### 4.1.2 Draw Spinning

Draw spinning, as an energy‐saving fiber spinning technique, is achieved via the stretching of a viscous polymer solution followed by solidification in air. For draw spinning, the spinning dope is required to exhibit elastic properties under low shear strain (around 1%) and viscous properties under high shear strain (around 1000%), which enables the dope to be elongated under stretching forces and facilitates the formation of free‐standing fibers. In light of the solidification mechanism, draw spinning can be divided into two categories: solvent evaporation‐induced solidification and phase separation‐induced solidification. In the subsequent sections, a detailed account of each will be presented.

**Figure 7:**
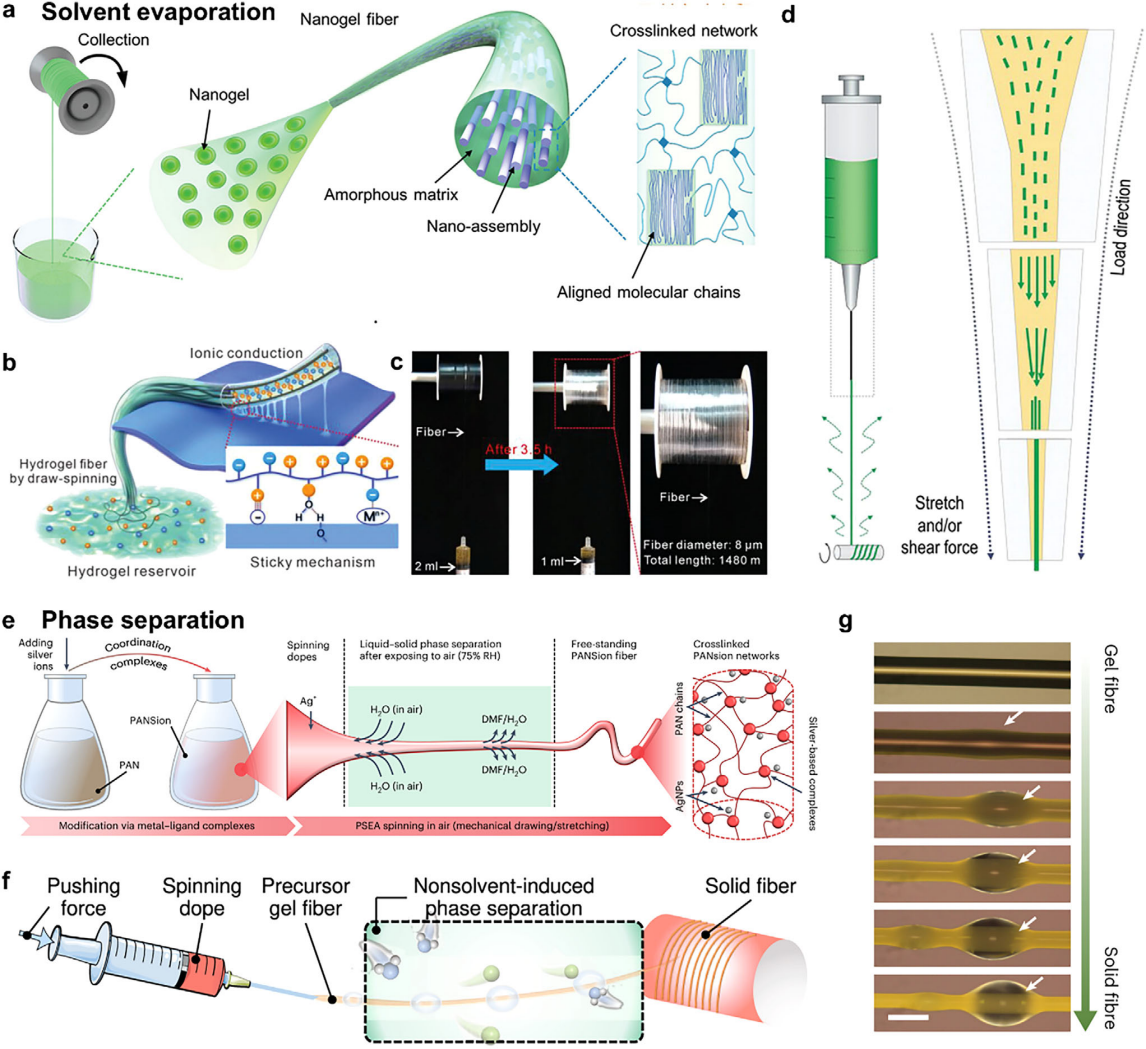
a) Schematic illustration of the spinning of the nanogel fiber. Reproduced with permission.^[^ ^112^ ^]^ Copyright 2022. Wiley‐VCH. b) Draw spinning of the PrDA hydrogel fiber for ion conduction and stickiness to the surface via a variety of interactions. c) Procedure of continuous draw‐spinning of P(VSA‐co‐DMAPAA) fiber. b,c) Reproduced with permission.^[^ ^95^ ^]^ Copyright 2023. Wiley‐VCH. d) Schematic illustration of our biomimetic spinning strategy. Reproduced with permission.^[^ ^96^ ^]^ Copyright 2023. Wiley‐VCH. e) The spinnable dope preparation using coordination complexes. f) Continuous fiber spinning via NVIPS spinning approach. Reproduced under the terms of the CC‐BY Creative Commons Attribution 4.0 International license (https://creativecommons.org/licenses/by/4.0/).^[^ ^113^ ^]^ Copyright 2023, published by Springer Nature. g) Optical microscopy images showing the fiber formation. The white arrows indicate the precipitated solvent droplets on the solid PANSion fiber. Scale bar, 50 µm. e,g) Reproduced with permission.^[^ ^97^ ^]^ Copyright 2023. Springer Nature.

The draw spinning of hydrophilic polymer solutions always involves water evaporation and the spinning solution solidification.^[^ ^110^, ^111^ ^]^ The spinning dope applicable for draw spinning has a high viscosity (>10^5^ Pa·s) and consists of high molecular weight polymers, such as polyacrylic acid (PAA, M_w_ ∼ 10^6^ Da), polyacrylamide (PAM, M_w_ ∼ 10^6^ Da), and branched polyethylene glycol (PEG, M_w_ ∼ 10^4^ Da). Attributed to the enhanced hydrogen bond nanoconfinement caused by water evaporation, a tough hydrogel fiber with a length of 2880 m and diameters in the range of 5–20 µm was obtained by drawing from a viscous solution containing polyacrylic acid nanogels and vinyl groups functionalized silica nanoparticles (**Figure** **7**a).^[^ ^112^ ^]^ Apart from hydrogen bonding interactions, the electrostatic interactions between polyelectrolytes can also be used to create ionic conductive fibers with high mechanical strength (> 100 MPa). For instance, Zhao et al.^[^ ^95^ ^]^ developed ionic conductive and sticky hydrogel fibers for signal transmission by employing electrostatic interactions based on proton donor‐acceptor sequences (Figure 7b,c). The proton transferred from the acid group of the donor to the basic group of the acceptor, thus forming electrostatic interactions between polyanions and polycations. This method can be applied to a wide range of proton donor and acceptor combinations with different dissociation constants (pKa), which significantly expands the polymer choices in fiber draw spinning.

Considering that the water evaporation should be conducted in an environment of low humidity (≈20% relative humidity), which needs a confined space and a dehumidifier, various types of volatile solvents like ether, acetone, and hexafluoroisopropanol (HFIP) are used to dissolve polymers for rapid evaporation in ambient environments. Specifically, a silk‐sourced ionotronic fiber was developed by pulling the silk microfibril/HFIP/Li^+^ solution with a liquid‐crystal‐like texture (Figure 7d).^[^ ^96^ ^]^

Another type of draw‐spinning approach that can fabricate ionic fibers in an ambient environment is to utilize phase separation. Zhang et al.^[^ ^97^ ^]^ fabricated stretchable, mechanically strong, and conductive fibers by drawing dope consisting of polyacrylonitrile (PAN) and silver ions dissolved in dimethylformamide (DMF) (Figure 7e). When the dope was drawn, the hygroscopic DMF absorbed water from the air, forming DMF/H_2_O solvent. Consequently, the solubility of PAN decreased, leading to the separation of the solid fiber from the solvent (Figure 7f,g).^[^ ^113^ ^]^ Such an energy‐saving spinning approach holds great potential for the sustainable manufacturing of ionic conductive fibers while paying more attention to solvent evaporation into the atmosphere and the high cost of solvent recycling.

Draw spinning enables the fast and continuous fabrication of ultrathin (≈10 µm) ionic conductive gel fibers with tunable compositions and high stretchability (>300% strain). This ambient‐pressure technique offers distinct advantages over conventional methods, including minimal solvent consumption, compatibility with roll‐to‐roll processing, and direct fiber integration into textile architectures. Nevertheless, critical limitations, such as solvent evaporation‐induced property degradation and reliance on hazardous solvents (e.g., HFIP, DMF) hinder long‐term stability and biocompatibility. To address these challenges, recent advances leverage hydrophobic surface encapsulation to mitigate dehydration and a hybrid core‐sheath design to balance ionic conductivity with mechanical resilience. These strategies position draw‐spun fibers as promising candidates for conformal bioelectronic interfaces and wearable sensors. Future efforts should be focused on green solvent alternatives (e.g., aqueous systems) and in situ stabilization methods to enable industrial‐scale wearable applications.

#### 4.1.3 Thermal Spinning

**Figure 8:**
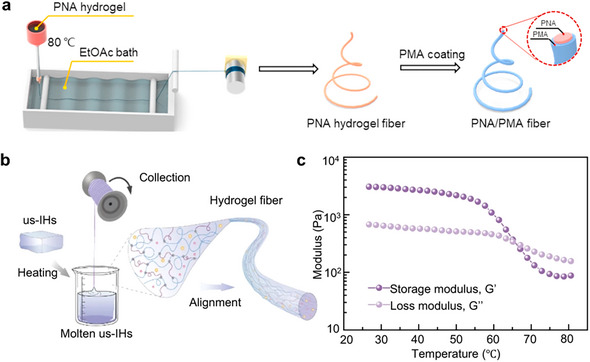
a) Schematic illustration of the preparation procedures of the PNA hydrogel fibers and PNA/PMA core‐sheath fibers. Reproduced with permission.^[^ ^98^ ^]^ Copyright 2020. Elsevier. b) Schematic illustration of the dip‐draw spinning of the ionic hydrogel fibers. c) Temperature‐dependent oscillatory rheology of us‐IHs (τ = 1%, ω = 10 rad s^−1^). b,c) Reproduced under the terms of the CC‐BY Creative Commons Attribution 4.0 International license (https://creativecommons.org/licenses/by/4.0/).^[^ ^99^ ^]^ Copyright 2024, published by Springer Nature.

Thermal spinning has been regarded as one of the most established and cost‐effective methods for fiber manufacturing in the industry. This approach takes advantage of the rapid cooling process to prompt the solidification of molten polymer. In contrast to commercial thermoplastic (e.g., nylon, polyethylene, polyester), which can be melted without the need for solvent, the production of ionic conductive hydrogel fibers involves the extrusion of molten polymer solution into cool air or bath to solidify the fibers. The existence of dynamic interactions within the polymer networks is the key factor in realizing the successful thermal spinning of ionic conductive fibers, which decompose under high temperatures and recover upon cooling.^[^ ^114^ ^]^ Shuai et al.^[^ ^98^ ^]^ fabricated stretchable, ionic conductive, and self‐healing hydrogel fiber by heating the physically cross‐linked poly (acrylamide‐co‐N‐acryloylglycinamide) (PNA)/LiCl hydrogel precursor at 80 °C and then extruding it into an ethyl acetate bath (**Figure** **8**a). The precursor exhibited a thermally reversible sol‐gel transition induced by the reversible formation and dissociation of hydrogen bonds at different temperatures.

Ionic conductive fibers with distinct viscoelastic behavior under different temperatures could be immediately molded at room temperature after dip‐drawing from the molten solution consisting of PVA, polyethyleneimine (PEI), and LiCl while avoiding the use of a large amount of coagulation bath (Figure 8b).^[^ ^99^ ^]^ The hydrogel transformed from elastic‐dominated behavior to viscous‐dominated behavior with the increase in temperature (Figure 8c). In addition, melt‐spun fibers are increasingly perceived as a global challenge in terms of resources, sustainability, and waste management. Continuous efforts to develop new types of sustainable fibers are necessary for a prosperous future.

The advantages of thermal spinning include high throughput, compatibility with thermo‐sensitive polymers (e.g., PNA, PEI, gelatin), and reduced environmental concerns compared to solvent‐based methods. However, challenges such as thermal energy input (≈80 °C), limited fiber uniformity (diameter variations >20%), and excessive inter‐fiber adhesion compromise process efficiency and downstream textile integration. Future efforts should focus on low‐melting‐point composites, in‐line diameter control systems, and in‐situ encapsulation strategies to bridge lab‐scale innovation and industrial wearable production.

#### 4.1.4 Direct Ink Writing

Direct ink writing (DIW) is an extrusion‐based additive manufacturing technique that utilizes a high‐precision deposition system to fabricate intricate architectures, including porous scaffolds, flexible electronics, and engineered biological tissues, through the layer‐by‐layer assembly of functional inks along predefined paths.^[^ ^115^ ^]^ Owing to its exceptional processing speed and scalability, DIW has recently emerged as a promising platform for high‐resolution (µm scale) and high‐throughput (km h^−1^ scale) fiber spinning.^[^ ^116^, ^117^ ^]^ The key point of this technology lies in the rational design of inks with suitable viscoelastic properties, where the viscosity exhibits shear‐thinning behavior to facilitate extrusion under shear force, followed by a rapid increase to preserve filament morphology after extrusion. To further improve the structural integrity for self‐support, several post‐processing strategies have been employed, encompassing: i) UV‐triggered photopolymerization for rapid covalent network formation,^[^ ^117^, ^118^ ^]^ ii) controlled thermal curing to optimize polymer chain entanglement,^[^ ^119^ ^]^ and iii) ionically mediated crosslinking strategies that leverage dynamic electrostatic interactions for self‐healing capabilities.^[^ ^120^ ^]^

DIW enables the precise fabrication of ionically conductive gel fibers, offering advantages such as customizable architecture, broad material compatibility, and scalable production for wearable ionic conductive textiles. However, challenges including stringent ink rheological requirements, limited mechanical durability, and complex post‐treating must be addressed through material optimization to ensure practical wearable applicability.^[^ ^121^ ^]^ Future advancements may focus on hybrid inks and seamless textile integration for robust and long‐term performance.

#### 4.1.5 Dynamic Crosslinking Spinning

**Figure 9:**
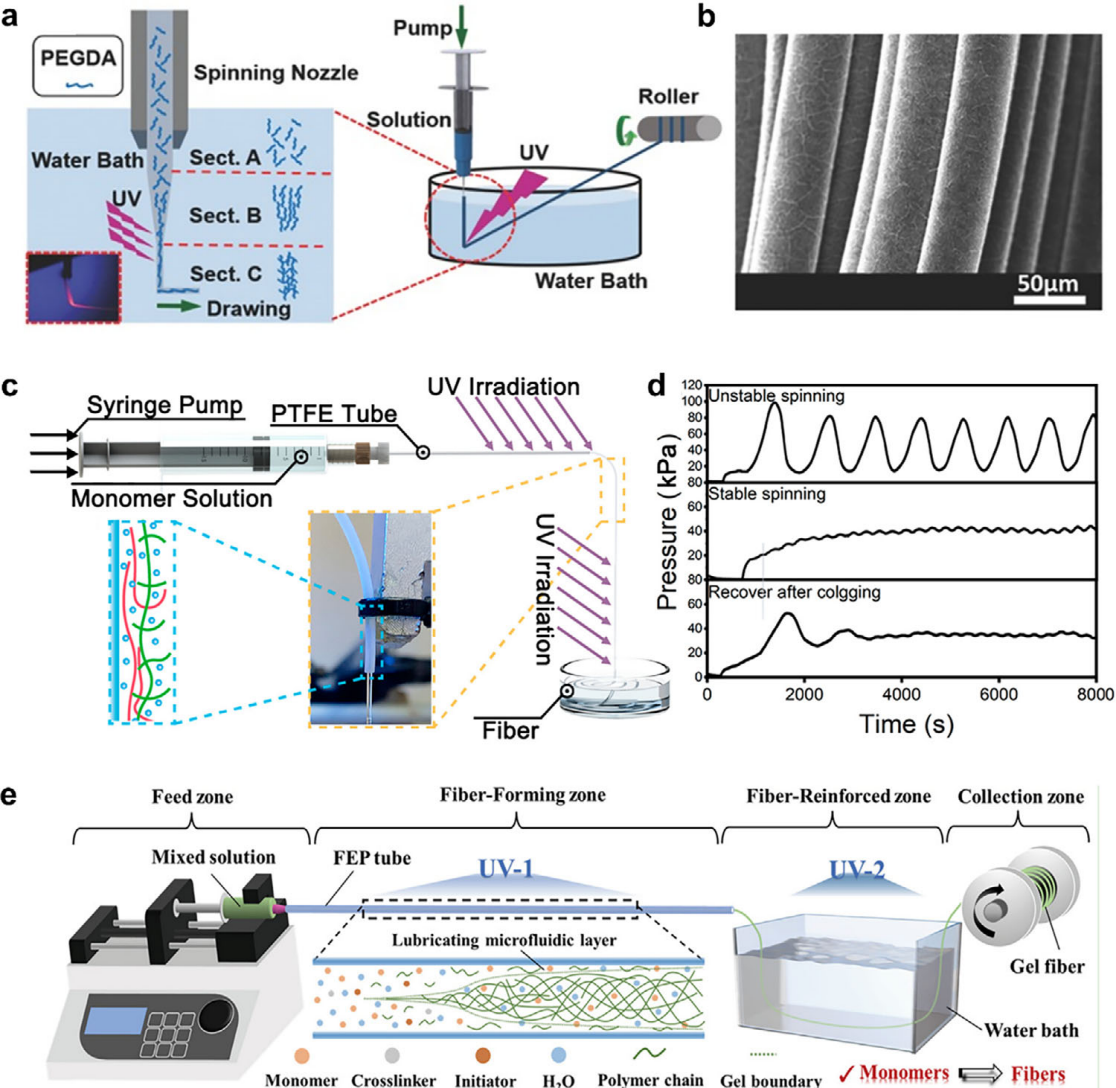
a) Schematic illustration of the dynamic‐crosslinking‐spinning. Enlarged schematic shows the polymerization process underdrawing force. Insert is the photo of the enlarged schematic (dyed by Rhodamine B). b) SEM images of a bundle of the hydrogel fibers. a,b) Reproduced with permission.^[^ ^122^ ^]^ Copyright 2016. Wiley‐VCH. c) Schematic illustration of the SLS apparatus. d) Experimentally measured pressure profiles during the different status of the spinning. c,d) Reproduced with permission.^[^ ^123^ ^]^ Copyright 2020. ACS. e) A continuous spinning system based on the SLS strategy. Reproduced under the terms of the CC‐BY Creative Commons Attribution 4.0 International license (https://creativecommons.org/licenses/by/4.0/).^[^ ^100^ ^]^ Copyright 2023, published by Wiley‐VCH.

Dynamic crosslinking spinning represents a novel method for the continuous and large‐scale fabrication of ionic conductive fibers. In this spinning process, a spinning solution containing monomer, solvent and photo‐initiator transforms from sol state to gel state, then solidifies into fibers via photoinitiated radical photopolymerization, allowing for the 100% utilization of raw materials without further post‐treatment. This method was first proposed by Zhu et al.^[^ ^122^ ^]^ to realize the successful large‐scale spinning of hydrogel fibers with uniform diameter utilizing the photopolymerization of poly(ethylene glycol diacrylate) solution (**Figure** **9**a,b).

The solidification of the solution generally needs to be fast and happen outside the nozzle to maintain the shape of the fiber and avoid tube block, which largely limits the wide applicability. To address this limitation, Duan et al.^[^ ^123^ ^]^ developed a self‐lubricating strategy in a dynamic crosslinking approach to ensure the smooth extrusion of partially polymerized gel fibers by isolating the gel from the tube wall with a thin lubricating layer of poly(2‐acrylamido‐2‐methylpropanesulfonic acid) (PAMPS) (Figure 9c). With the addition of the lubricating layer, the extrusion pressure became stable (Figure 9d).

The PAMPS layer restricted the selection of the monomer due to the compatibility problem. To expand the universality of monomers in the fiber design strategy, the lubricating layer was introduced by using a hydrophobic tube, such as fluorinated ethylene propylene, silica gel, and polytetrafluoroethylene (Figure 9e).^[^ ^100^ ^]^ The lubrication effect was attributed to the oxygen being trapped in the interface between the monomer solution and the rough hydrophobic material tube. The salts can also be incorporated into the spinning dope or solvation bath to endow the fiber with excellent ionic conductivity.^[^ ^101^ ^]^

Dynamic crosslinking spinning offers broad monomer and ion compatibility, enabling the fabrication of ionic conductive fibers via photoinitiated polymerization under mild conditions and eliminating the need for high temperatures or harsh solvents. Nevertheless, precise control over crosslinking kinetics during high‐speed spinning remains challenging due to sluggish polymerization rates (>10 s for solidification), significantly hindering production scalability. Future research should prioritize accelerating spinning speeds and developing recyclable/respinnable systems to advance sustainable manufacturing.

#### 4.1.6 Electrospinning

**Figure 10:**
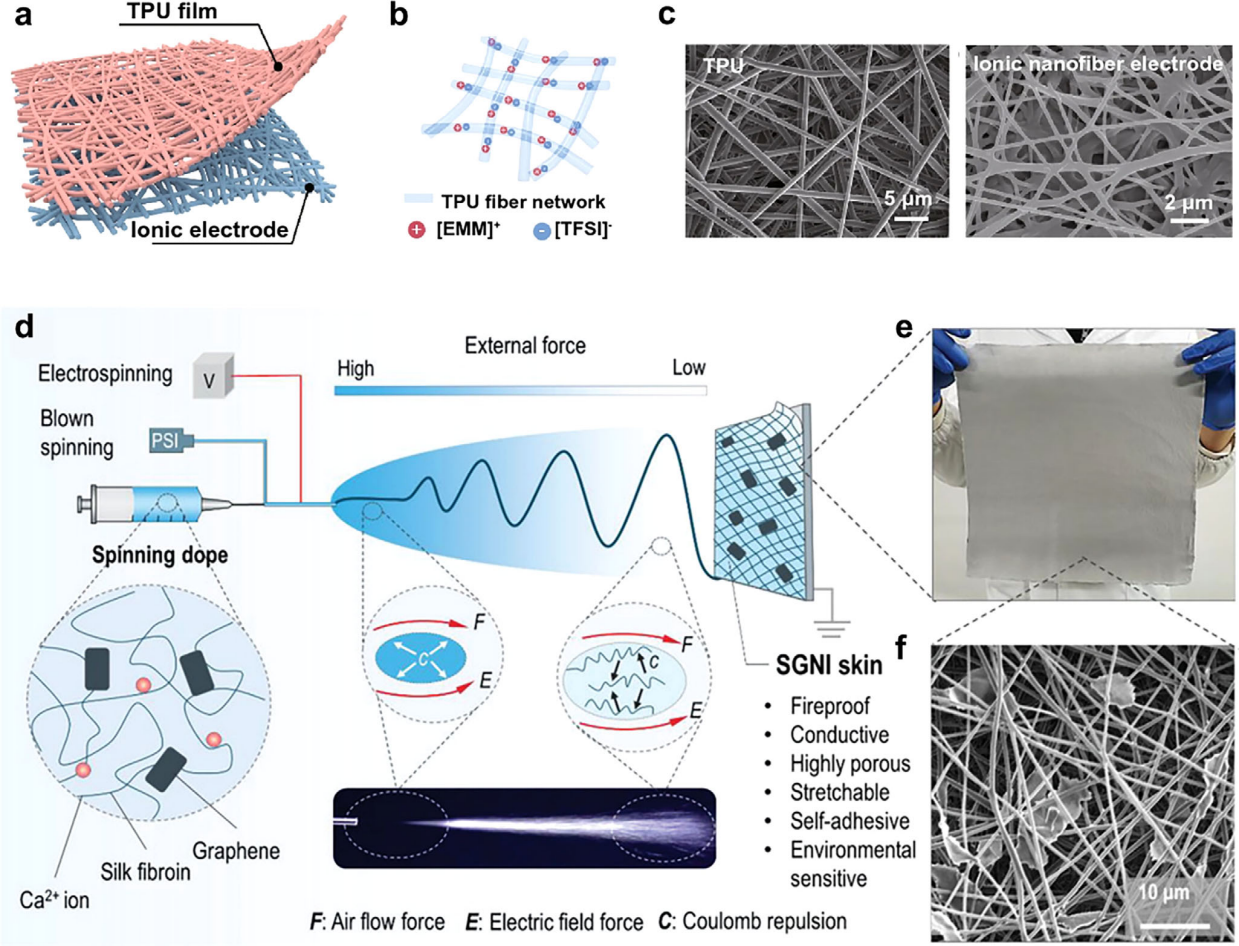
a) Basic structure scheme of the iontronic triboelectric mechanoreceptor. b) Conductive mechanism of the ionic nanofiber electrode based on TPU scaffold and the ionic liquid. c) SEM images for pure TPU nanofibers and the ionic nanofiber electrode (ionic liquid of 60 wt%). a,b,c) Reproduced under the terms of the CC‐BY Creative Commons Attribution 4.0 International license (https://creativecommons.org/licenses/by/4.0/).^[^ ^102^ ^]^ Copyright 2022, published by Springer Nature. d) Schematic for the fabrication process of protection skins. e) The SGNI skin with dimensions of 30 cm × 30 cm. f) Microstructure of the SGNI skin. d,e,f) Reproduced with permission.^[^ ^125^ ^]^ Copyright 2021. Wiley‐VCH.

Electrospinning, an emerging technology that has begun its transition from the laboratory‐based incubation stage to commercial‐scale manufacturing, has great potential for developing polymer nanofibers with porous microstructures under low processing temperatures. The setup for electrospinning consists of a spinneret connected with a high‐voltage power supply and a rotating conductive collector. When ions or polyelectrolytes are incorporated into the spinning dope, the resultant porous fiber mat can be ionic conductive to form a fibrous dielectric layer of the capacitor, an ionic electrode of the triboelectric generator, and a resistive layer of the resistor. To achieve both good air permeability and stable sensing capacity in a moist environment, a hydrophobic poly(ionic liquid) was electrospun into fibrous membranes with rich ions and microporous structures, which was ideal for dielectric materials for stable signals sensing under moisture interference.^[^ ^103^ ^]^ Besides, the polymer chain network of nanofiber could offer a homogeneous medium allowing highly mobile ions confined within the nanofibers, thus ensuring excellent conductivity (**Figure** **10**a,b).^[^ ^102^, ^124^ ^]^ With the addition of ionic liquid, the nanofiber became cohesive to provide excellent adhesion with other functional layers and human skin (Figure 10c). From the perspective of sustainability, biopolymers that have high biocompatibility, biodegradability, and recyclability are also selected to fabricate nanofibrous fabrics. Cao et al.^[^ ^125^ ^]^ developed a silk/Ca^2+^/graphene ionotronic skin with stretchability up to 400% through an electro‐blown spinning technique (Figure 10d).

Ionic conductive fiber mats with high porosity, tunable fiber diameter, and large surface area can be fabricated through electrospinning, which is ideal for wearable ionotronics. However, challenges include limited ionic conductivity and difficulty in achieving large‐size textiles, which may affect the performance of wearable devices. Despite these drawbacks, electrospun ion‐conductive fibers remain promising for wearables due to their lightweight nature, breathability, and compatibility with stretchable substrates, making them suitable for applications like E‐textiles and bioelectrodes.

### 4.2 Engineering Fibers into Yarns

Fibers are micro/nanoscale individual units, while yarns are macroscopic assemblies of fibers that enhance mechanical robustness and ion transport efficiency. The engineering of ionic conductive fibers into functional yarns is critical because: i) Interconnected networks in yarns reduce interfacial resistance, boosting ionic conductivity; ii) Structural resilience (e.g., braided or helical designs) prevents fracture under strain, maintaining functionality; iii) Textile compatibility enables weaving/knitting for wearables; and iv) Multifunctionality allows hybrid designs (e.g., core‐sheath fibers for dual ion/electron transport). Thus, yarn engineering bridges nanoscale material properties and macroscale applications in flexible electronics and energy storage.

#### 4.2.1 Twisting

Twisting, one of the facile assembly methods that involves rotating a single fiber along its central axis or helically coiling several fibers to form a yarn, enhances inter‐fiber contact by promoting surface interactions or facilitating electrolyte infiltration between filaments.^[^ ^127^ ^]^ The increased contact area potentially facilitates the formation of ionic pathways. Leveraging this principle, integrating twisted structures into ionic conductive fibers has enabled the development of high‐performance artificial muscles,^[^ ^128^, ^129^ ^]^ high‐energy harvesting efficiency triboelectric nanogenerators,^[^ ^130^ ^]^ and ultrasensitive strain sensors^[^ ^131^ ^]^ with enhanced strength, toughness, and sensing performance. Higher twisting degree reduces the distance between fibers, thereby increasing the effective contact area for enhanced charge transmission. Furthermore, such highly twisted architectures enable strain redistribution, effectively mitigating localized deformations during fiber stretching, which is a critical feature for maintaining structural integrity under mechanical stress.

#### 4.2.2 Helical Winding

Helical winding offers an alternative approach, where conductive filaments are spirally wrapped around a core fiber, forming continuous conductive loops that enable long‐range ion transport.^[^ ^132^ ^]^ This architecture is particularly advantageous for stretchable electronics with high robustness, as the helical geometry accommodates strain without sacrificing performance.^[^ ^133^ ^]^ For example, Feng et al. developed an all‐in‐one capacitive yarn sensor featuring a coaxial helical architecture, fabricated through the precise helical winding of conductive electrodes around an elastic ionogel fiber.^[^ ^134^ ^]^ The helical electrode configuration maintains stable, high conductivity under mechanical deformation (>100% strain), while the ionogel fiber core significantly enhances sensing performance via the substantial electric double‐layer capacitance at the electrode‐ionogel interface.

#### 4.2.3 Braiding

Braiding, a more complex 3D interlacing technique where multiple fibers or yarns are woven into tubes or strips in an interleaved spiral pattern, provides mechanical redundancy and multiple conductive pathways, ensuring stability even under fiber breakage.^[^ ^135^ ^]^ The interconnected pores in channeled structures can be leveraged for electrolyte penetration and dispersion, significantly improving ion mobility, especially advantageous in fiber batteries. For instance, Huang et al. engineered a multi‐channel braided fiber current collector via multi‐axial winding, significantly enhancing active material loading while facilitating rapid ion transport.^[^ ^136^ ^]^ The optimized design realized a fiber battery with an exceptional energy density of 62 Wh kg^−1^, demonstrating the synergistic benefits of structural porosity and conductive pathways in wearable energy storage systems.

### 4.3 Integration of Ionic Conductive Textiles

Although a single ionic conductive fiber could realize the functionalities of sensing, luminescence, and health monitoring, attaching it to human skin is still in the early stages of E‐textiles. The further treatment for wearing comfort and system integration is to fabricate ionic conductive fabrics by using the following methods: i) weaving/knitting/knotting intrinsically ionic conductive fibers into fabrics; ii) coating ionic conductors onto textiles.

#### 4.3.1 Integrating Ionic Conductive Yarns into Fabrics

**Figure 11:**
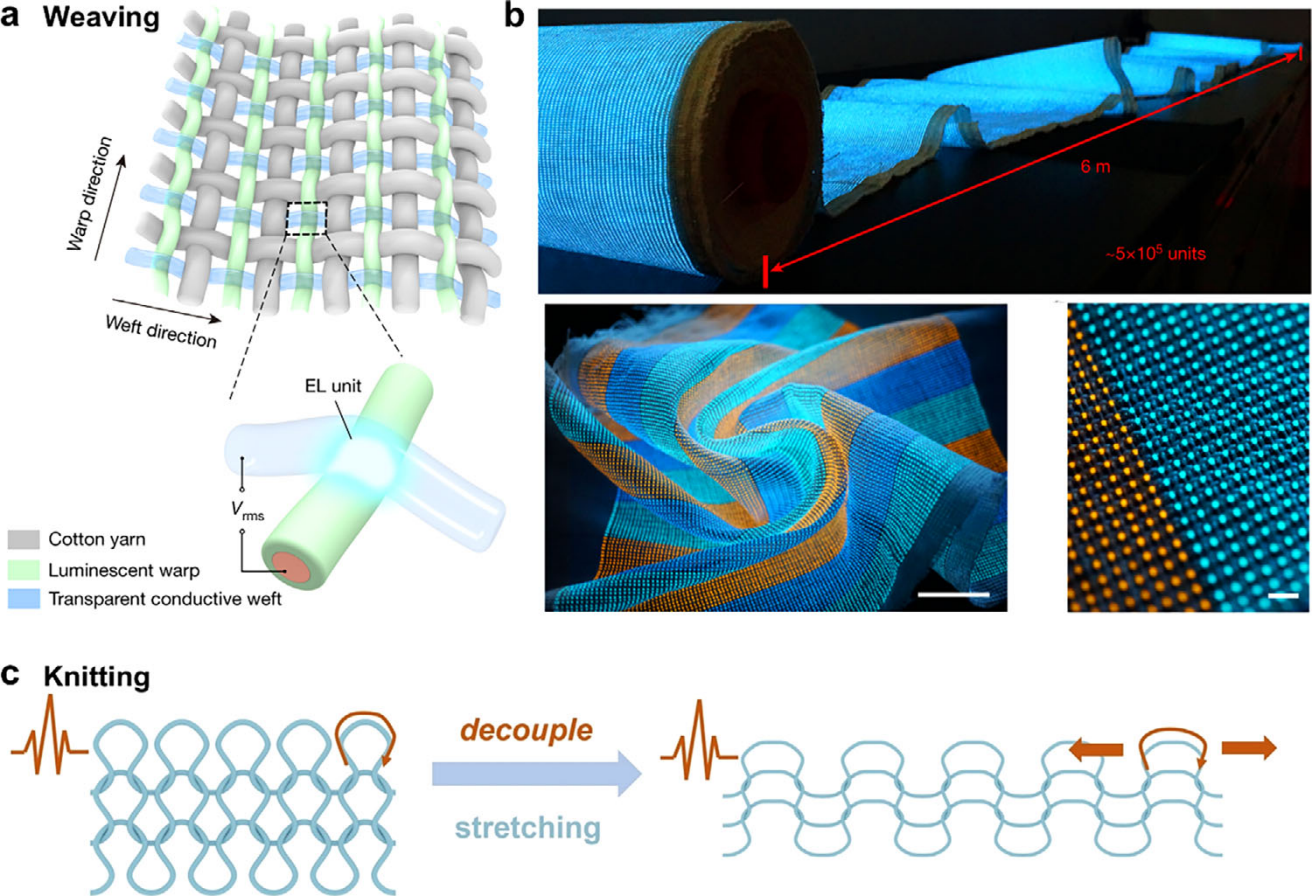
a) Schematic showing the weave diagram of the display textile. Each contacting luminescent warp and transparent conductive weft forms an EL unit (inset). b) Photograph of a 6‐m‐long display textile consisting of approximately 5 × 10^5^ EL units. Photograph of a functional multicolor display textile under complex deformations, including bending and twisting and magnified photograph of the multicolor display textile shows that the EL units are uniformly spaced at a distance of ≈800 µm. Scale bar, 2 mm. a,b) Reproduced with permission.^[^ ^66^ ^]^ Copyright 2021. Springer Nature. c) Mechanism of the topological pattern to enhance the stretchability. Reproduced with permission.^[^ ^82^ ^]^ Copyright 2023. Wiley‐VCH.

Ionic conductive fabrics can be crafted via weaving, knitting, and knotting, which are conventional textile manufacturing techniques to convert yarns into fabrics. Weaving involves creating a fabric from two sets of interlaced yarns: the lateral weft yarns and the longitudinal warp yarns. In woven fabrics, the extensive interlacement within the woven structure imparts firmness and stability, which is especially valuable in display textiles, where it can construct electroluminescent units. For example, Shi et al.^[^ ^66^ ^]^ chose an ionic liquid‐doped polyurethane gel as the transparent conductive weft and a zinc sulfide (ZnS) phosphor‐coated conductive yarn as the luminescent warp (**Figure** **11**a). The weaving process enabled the production of a large‐area display textile measuring 6 m × 25 cm (length x width), containing around 5 × 10⁵ EL units (Figure 11b). Furthermore, in electroluminescent devices, weaving enables the interaction between phosphors and ionic electrodes through cross‐overlap, as opposed to the multi‐layer coating approach. Through weaving, issues such as inconsistent display performance, device failure and accidental short‐circuit can be avoided since coatings on the surfaces of 1D fibers are prone to being highly non‐uniform when compared to 2D planes due to the relatively high surface tension of the 1D fibers and the unintentional flow of the coating material under the influence of gravity. Considering the interlaced structure of warp and weft yarns in weaving, the fabric may have limited flexibility in certain directions compared to knitted fabrics and potential for yarn damage during weaving.

On the other hand, knitting encompasses a range of textile structures formed by interlacing yarns. The characteristic meandering loops in knitted fabrics endow them with remarkable stretchability. Significantly, this stretchability can be achieved even when the constituent yarns of fabrics are not inherently stretchable. For instance, Deng et al.^[^ ^82^ ^]^ functionalized natural cotton (stretchability of around 12%) with poly (3,4 – ethylenedioxythiophene): poly (styrene sulfonate) (PEDOT: PSS) and then knitted it into a highly stretchable fabric (stretchability of around 300%) (Figure 11c). The interspace among the numerous aligned nanofibrils in cotton fibers was also exploited as nanofluidic channels for ion and electron conduction. Since knitting can transform materials with poor stretchability, such as functionalized natural cotton, into fabrics with high stretchability, it offers more flexibility in the selection of raw materials when fabricating ionic conductive fabrics. Stiff fibers with limited stretchability can be used to facilitate the development of ionic conductive textiles.

Knotting is a textile fabrication technique where fibers or yarns are interlocked through knots (e.g., square knots, figure‐eight knots) to form flexible and porous structures. It is widely used in traditional crafts, fishing nets, and medical sutures, and has recently been adapted for smart textiles due to its tunable mechanical and conductive properties. Leveraging the distinct strain‐responsive dynamics of knots, a suite of high‐performance electromechanical sensors through adaptive structural optimization was developed.^[^ ^137^ ^]^ The reef knot configuration enabled a strain sensor with a gauge factor of 6.5 at 7.6% elongation, while double overhand knots formed pressure‐sensitive architectures. Notably, clove hitch knot geometries endowed ultra‐responsive sensors with a 57 ms activation time, demonstrating how topological control of ionic pathways can tailor device performance metrics. Moreover, the knotted structure can be applied in the field of textile‐based actuators and fiber transistors for higher flexibility.^[^ ^123^, ^138^ ^]^

With the development of technology and the demand for large‐scale textile manufacturing, conventional handicraft methods were gradually replaced by industrial production. The handicraft methods rely on workers manually using tools (e.g., rods, crochet, and shuttles) to integrate fibers into textiles, which is a slow process but suitable for personalized customization with small‐batch production. The manual weaving process of ionic conductive fibers requires only simple tools, such as a handloom, without the need for expensive equipment.^[^ ^139^ ^]^ In contrast, industrial textile manufacturing relies on automated machines for mass production, offering high efficiency, uniformity, and lower costs but limited design flexibility. In the abovementioned ionic conductive textiles, the weaving operation of display textiles was realized by an automated loom,^[^ ^66^ ^]^ and the actuator textiles were knitted in plain patterns using a knitting machine.^[^ ^65^ ^]^ When weaving and knitting, the strength of fibers should be high enough to withstand the tension, and the surface stickiness of fibers should be eliminated to reduce friction. To enable scalable manufacturing of ionic conductive textiles, future research must transition from labor‐intensive manual fiber weaving to automated machine weaving techniques.

#### 4.3.2 Coating Ionic Conductors onto Textiles

**Figure 12:**
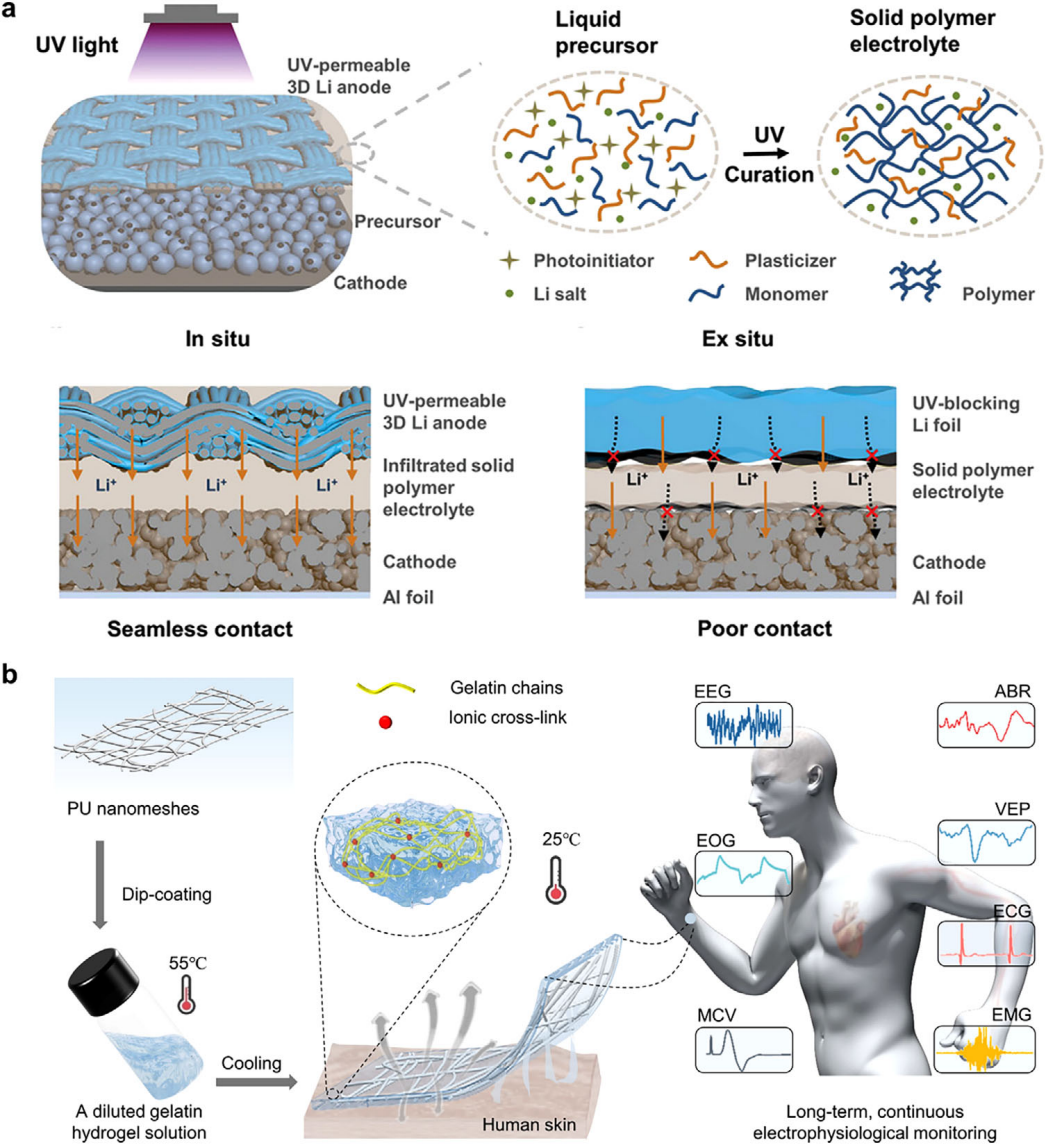
a) Schematic illustration showing in situ fabrication of SSLMBs and polymerization of liquid precursor under UV irradiation. Smooth Li^+^ transport enabled by seamless interfaces of the in situ fabricated cell. Poor Li^+^ transport due to the large gaps of the ex situ‐assembled cell. Reproduced under the terms of the CC‐BY Creative Commons Attribution 4.0 International license (https://creativecommons.org/licenses/by/4.0/).^[^ ^142^ ^]^ Copyright 2024, published by Wiley‐VCH. b) Schematic illustration of the design concept of PU nanomesh‐reinforced hydrogels used for long‐term, continuous electrophysiological monitoring. Reproduced under the terms of the CC‐BY Creative Commons Attribution 4.0 International license.^[^ ^143^ ^]^ Copyright 2024, published by AAAS.

Apart from constructing ionic conductive textiles using ionic conductive fibers as building blocks, the assembly of ionic conductors with commercial or electrospun textiles is also a promising way to impart ionic conductivity to dielectric textiles. One method is to coat ionic conductors onto fabrics. Zhang et al.^[^ ^140^ ^]^ have coated ionic PVA/NaCl hydrogel onto carbon black modified fabric to form an asymmetric hygroscopic structure that could simultaneously achieve energy harvesting and storage from ambient moisture absorption. Another strategy is to develop an ionic textile (i‐textile) with good water resistance and sensing capabilities through in situ photopolymerization of ionogel onto the textile surface.^[^ ^141^ ^]^ The i‐textile presents considerable air permeability comparable to that of bare textiles while possessing enhanced ultraviolet (UV resistance. This strategy can also be utilized in the energy field, like flexible solid‐state lithium metal batteries. Xie et al.^[^ ^142^ ^]^ proposed a one‐step integration of the cathode, anode, and solid electrolyte via an in situ UV‐initiated polymerization (**Figure** **12**a). The UV irradiation could penetrate the UV‐permeable 3D Li anode to initiate the polymerization of polymer electrolytes, thus eliminating the interfacial gaps between the electrodes and the solid electrolytes to regulate the Li plating/stripping behaviors.

Another method to combine ionic conductors with textiles is incorporating elastic nanomesh into ionic gel to develop ultrathin and comfortable hybrid ionic fabrics. Wang et al.^[^ ^144^ ^]^ fabricated ionic sensing fabrics that could balance the trade‐off between fatigue resistance and self‐healing ability, originating from the ion‐rich conductor and hierarchically arranged nanofibrous structure like human skin. When stretched, the amorphous nanofiber would rearrange to a highly ordered state to enhance the stiffness and fatigue resistance to crack propagation. Furthermore, Zhang et al.^[^ ^143^ ^]^ presented a 10‐micrometer‐thick polyurethane nanomesh‐reinforced gelatin hydrogel sensor with excellent gas permeability and ionic conductivity for continuous electrophysiological monitoring (Figure 12b). The nanomesh polymer network provided a backbone and toughening agent for the free‐standing of ultrathin composites, while the ionic gels endowed composites with great ionic conductivities for stain‐sensing and health‐monitoring.

## 5 Applications

Due to excellent properties such as tunable mechanical performance, high ionic conductivity, and biocompatibility, ionic conductive textiles have found extensive use in a wide range of applications. In this part, the applications of ionic conductive textiles in sensors, energy harvesters and storage, and ionic processors are summarized. Moreover, the functions of ionic conductors in devices are presented, accompanied by the working mechanisms and performance of devices.

### 5.1 Sensors

**Figure 13:**
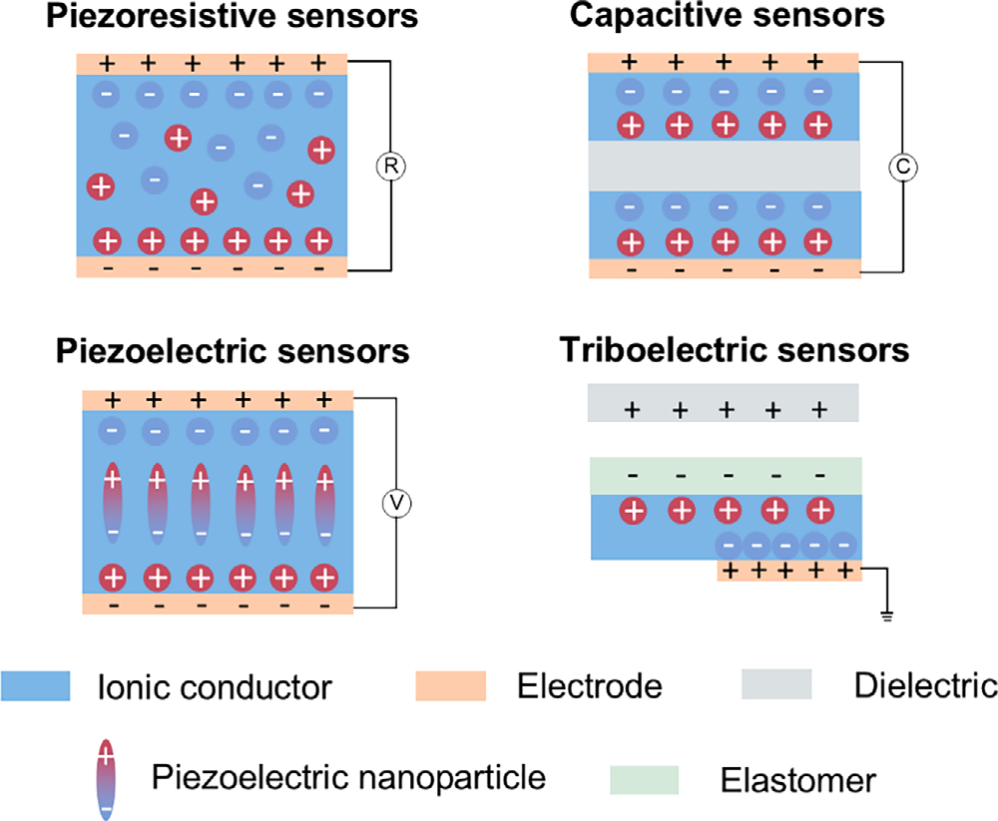
Different types of strain and pressure sensors, including piezoresistive, capacitive, piezoelectric, and triboelectric sensors.

Wearable sensors have emerged as a transformative technology with the potential to significantly impact human life, particularly in the realms of healthcare monitoring. These devices provide continuous, real‐time, and non‐invasive tracking of mechanical stimulation. According to the sensing mechanism, pressure and strain sensors can be categorized as piezoresistive, capacitive, triboelectric, and piezoelectric types (**Figure** **13**). These sensors can convert mechanical stimulation into corresponding changes in resistance, capacitance, electric field, and voltage to realize sensing. This section discusses the material selection and structural design of fiber‐based pressure and strain sensors according to these four types of sensing mechanisms.

#### 5.1.1 Piezoresistive Sensors

A piezoresistive sensor operates on the principle that the resistance of ionic conductors varies in response to applied pressure or strain, enabling the determination of pressure or strain by analyzing the corresponding changes in resistance. Fibers are highly amenable for use as sensing layers in piezoresistive sensor fabrication. Their advantageous flexibility, along with the ability to offer ample scope for resistance changes, greatly enhances the detection range of sensors. For example, Simon et al.^[^ ^145^ ^]^ prepared electrospun fibers from polyvinylidene fluoride that incorporated graphene nanoplatelets through a noncovalent functionalization technique, utilizing 1‐butyl‐3‐methylimidazolium trifluoromethanesulfonate ionic liquid. This approach aimed to develop an electroactive material suitable for pressure‐sensing applications. The resulting pressure sensor exhibited superior performance, characterized by minimal hysteresis and a narrower distribution of measured values, particularly within the operational range of 0 to 250 kPa. Furthermore, the piezoresistive response of the sensor was evaluated over 32 load‐unload cycles, demonstrating consistent stability throughout the tests. Sun et al.^[^ ^146^ ^]^ synthesized ionogel fibers through in situ radical polymerization. The fabricated ionogel fibers showed outstanding anti‐freezing properties. Even at a low temperature of −20 °C, the fibers could stretch by over 300% and maintain conductivity at 0.24 ± 0.09 mS cm^−1^. In addition, these ionogel fibers could be smoothly integrated with standard fabrics. As a result, they are suitable for use as strain sensors to monitor human motion.

#### 5.1.2 Capacitive Sensors

The capacitive sensor detects the mechanical stimulation through variations in its capacitance. Typically, these sensors are composed of two conductive electrodes separated by a dielectric material. When an alternating current is applied, opposite charges gather on the upper and lower electrodes. Consequently, the capacitance can vary with alterations in geometry during loading and unloading, independent of the resistance of the fiber electrodes. As the sensor is stretched or compressed, both the area of the electrodes and the thickness of the dielectric layer vary with the device structure, leading to a corresponding change in capacitance. For example, Feng et al.^[^ ^134^ ^]^ developed an innovative waterproof ionotronic all‐in‐one capacitive yarn sensor to boost sensitivity for various elongation strain measurements. The construction involved helically wrapping a flexible, non‐stretchable conductive wire around a rubber elastic cord, which serves as the core electrode. Following this, ionic gels were applied to the core electrode to secure its position, leading to the formation of an inner core fiber. This capacitive yarn sensor can be easily integrated into daily clothing, enabling the monitoring of human movements and physiological data like respiration, heart rate, and pulse.

#### 5.1.3 Piezoelectric Sensors

Piezoelectric pressure sensors can be constructed using piezoelectric substances, including zinc oxide (ZnO), barium titanate (BaTiO_3_), and poly(vinylidene fluoride) (PVDF). Typically, a piezoelectric sensor consists of conductive electrodes with a piezoelectric sensing layer positioned between them. When mechanical stimulation is applied to a piezoelectric material, it leads to a deformation of its crystalline structure, resulting in the displacement or separation of charges. When a piezoelectric material reverts to its original state, equal positive and negative charges form on opposite surfaces, generating electrical pulses (voltage or current). This creates an electric field or voltage across the material, known as the piezoelectric effect. For instance, Villa et al.^[^ ^147^ ^]^ fabricated a piezoionic/piezoelectric polymeric nanocomposite based on a chemically cross‐linked ionogel containing BaTiO_3_ nanoparticles. The composite can respond to low‐frequency (0.1–1 Hz) mechanical compressive stresses in the low‐pressure range (<10 kPa), generating output voltages of up to 8 mV.

#### 5.1.4 Triboelectric Sensors

The working principle of triboelectric sensors is based on the interplay between triboelectric charging and electrostatic induction. To construct these sensors, it is essential to combine two polymers with different electron energy levels, thereby forming a friction pair. Given that nearly all polymers exhibit the characteristic of frictional electrification, triboelectric sensors benefit from a wider array of device configurations and a more diverse selection of materials. All layers in triboelectric sensors, including triboelectric layers and electrodes, can be shaped into fiber form. For example, Sheng et al.^[^ ^148^ ^]^ prepared an organogel/silicone fiber‐helical sensor based on a triboelectric nanogenerator (OFS‐TENG) for power‐free and sutured implantation of ligament strain monitoring. The helical inner core of the OFS‐TENG consists of an organogel fiber (serving as a conductive electrode) and a silicone fiber (functioning as a dielectric layer). Within the sensor, triboelectrification and electrostatic induction jointly occurred when the two fibers contacted and released. This design enables sensitive monitoring of movements in ligaments, muscles, and surrounding soft tissues.

### 5.2 Energy Harvesters

Ionic conductors‐based fabrics with energy harvesting capabilities are of great attention in textile technology, allowing the harvesting of energy from the daily movement of the human body or the resources of the environment. These ionotronic fiber‐based energy harvesters pave the way for the next generation of renewable energy, allowing for sustainable energy utilization.

#### 5.2.1 Triboelectric Generators

**Figure 14:**
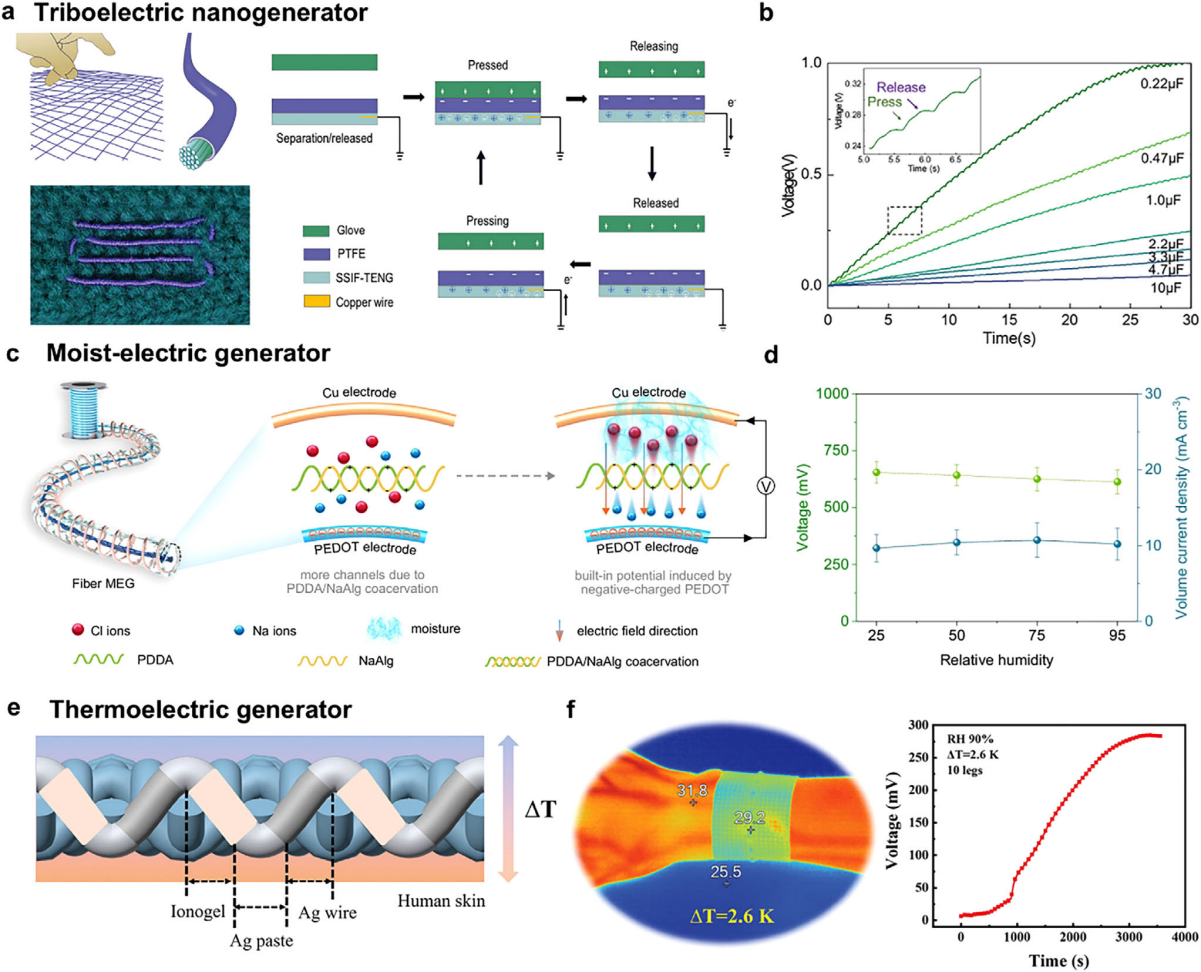
a) Schematic illustration of SSIF‐TENG in the single‐electrode mode and corresponding circuit diagram in response to a deformation. Working principle of SSIF‐TENG in single‐electrode mode. b) Measured voltages of different capacitors charged with SSIF‐TENG at 2 Hz. a,b) Reproduced with permission.^[^ ^96^ ^]^ Copyright 2023. Wiley‐VCH. c) Schematic of synergetic strategy of complex coacervation and built‐in potential. d) PEDOT@P5N20 fibers at different RH values. c,d) Reproduced under the terms of the CC‐BY Creative Commons Attribution 4.0 International license (https://creativecommons.org/licenses/by/4.0/).^[^ ^151^ ^]^ Copyright 2024, published by Springer Nature. e) Schematic diagram of the wearable FITED fabricated by weaving the ionogel fiber into a fabric. f) Temperature gradient and thermovoltage generated when wearing the FITED. e,f) Reproduced with permission.^[^ ^78^ ^]^ Copyright 2024. Wiley‐VCH.

A triboelectric generator (TENG) generates the voltage through the repeating contact‐release cycles between triboelectric layers with different charges. TENG holds great promise for integration with textiles to harvest energy from human body motions or friction during exercise.^[^ ^149^, ^150^ ^]^ Ionic conductive fibers serve as the electrode layer, which collects and conducts the charges generated by the triboelectric layers, enabling the formation of electrical current output. Compared to metal electrodes, ionic conductors preserve structural integrity and electrical performance even under the stretching and bending motions of the human body, which ensures the stable operation of TENG. Cao et al.^[^ ^96^ ^]^ fabricated a textile‐based TENG by weaving core‐shell structured ionic conductive fibers with commercial yarns (**Figure** **14**a). The electrons flowed in different directions when the glove yarns contact or separated from TENG fibers, thus generating triboelectricity. The electric energy produced by the TENG textiles can be stored in capacitors using a rectifier (Figure 14b). In addition, the generated voltage signals can also be used for tactile perception, as discussed in 5.1.4. Besides tactile sensing, the hydrogel‐based TENG patch, integrating an ionic conductive hydrogel textile, conductive wiring, and an adhesive substrate, was designed to harvest biomechanical energy and deliver programmable electric fields to damaged tissues.^[^ ^20^ ^]^ In vitro studies demonstrated that the TENG‐generated electric potential significantly enhances fibroblast migration, proliferation, and paracrine secretion of angiogenic factors in both healthy human dermal fibroblasts and diabetic‐derived fibroblasts, demonstrating the potential in biomedical applications.

#### 5.2.2 Moist‐Electric Generators

Moist‐electric generators have emerged as a promising solution for harvesting electricity from ambient moisture, which offers a sustainable and potentially widespread source by converting the energy present in moisture into electrical energy. Ionic conductors in moist‐electric generators typically are composed of hygroscopic salts, responsible for absorbing moisture and ion conduction. The moist‐electric generators operate on a principle related to the ion concentration gradient realized by an asymmetric structure design. Zhang et al.^[^ ^140^ ^]^ constructed a moisture‐driven energy generator through the asymmetric deposition of a hygroscopic ionic hydrogel onto a layer of functionalized carbon fabric. The interaction between the hygroscopic hydrogel and the underlying carbon fabric induces an in‐plane potential difference of over 0.6 V between the wet and dry regions of the carbon fabric. Moreover, the asymmetric design can be extended into a core‐shell structure fiber to increase the flexibility of the generators. For example, Zan et al.^[^ ^151^ ^]^ developed a high‐performance uniaxial moisture‐driven electricity generator, which has a poly(3,4‐ethylenedioxythiophene) (PEDOT) core with a built‐in charge potential and a gel shell made of a poly(diallyldimethylammonium chloride) (PDDA) and sodium alginate (NaAlg) coacervate (Figure 14c). During coacervation, strong polymer‐polymer assembly increased the free volume and released numerous mobile ions, leading to more charge carriers and faster ion diffusion. Notably, the uniaxial fiber‐based moist‐electric generator achieved an output voltage of up to 0.8 V, a maximum current density of 1.05 mA cm^−2^, and a power density of 184 µW cm^−2^ at 20% relative humidity, alleviating the problem of low power generation efficiency of moist‐electric generator in a dry environment (Figure 14d).

#### 5.2.3 Thermoelectric Generators

Among various technologies, fiber‐based thermoelectric generator devices capable of harvesting waste heat in the human body and converting heat into electricity are burgeoning, spurred by concerns over energy consumption and climate disasters. Despite numerous efforts dedicated to enhancing the performance of electron‐based thermoelectric materials, their low thermo‐voltage (S < 100 µV K^−1^) and the relatively low‐grade waste heat of the human body (∆T < 10 K) pose challenges in powering small devices. Ionic thermoelectric materials, employing ions as charge carriers, have garnered growing interest owing to their higher thermovoltage compared to electronic thermoelectric materials, a result of their high ionic Seebeck coefficient and ionic conductivity. When there is a temperature gradient, the diffusion, migration, and dissociation of ions will change, resulting in an ion concentration gradient, which in turn forms an electric potential difference to achieve thermoelectric conversion. Li et al.^[^ ^78^ ^]^ fabricated a long ionogel fiber with ten pairs of p‐type units connected in series to construct a wearable ionogel fiber‐based ionic thermoelectric generator device (Figure 14e). Leveraging the temperature difference between the human body and the environment (∆T = 2.6 K), wearing the device on the arm generates a thermovoltage of 284 mV, as depicted in Figure 14f.

### 5.3 Energy‐Storage Devices

Ionic conductors, serving as electrolytes in both supercapacitors and batteries, are important for the power supply of wearable devices. The larger specific surface area is provided by ionic conductive textiles, which enhances the performance of devices but also renders feasibility for seamless integration into fabrics. Such a large surface area facilitates more efficient charge transfer and interaction processes within the devices, thereby leading to improved performance. Moreover, the seamless integration into fabrics enabled by these textiles holds great promise for various applications, such as wearable electronics, where wearability and flexibility can be achieved without compromising functionality.

#### 5.3.1 Supercapacitors

The operation of supercapacitors, including fiber‐based ones, depends on the ionic conductors for ion transfer (in electrostatic double‐layer models) or to participate in chemical reactions (in pseudocapacitance models).^[^ ^152^ ^]^ The ionic conductors need to form a stable interface with the electrode, which is conducive to efficient charge transfer. Ionic conductive textiles mostly function as solid and gel electrolytes in supercapacitors. The ionic conductors facilitate the rapid charge and discharge processes, allowing for the efficient movement of ions between the electrodes, which is vital for the fast storage and release of electrical energy. In addition, the ionic conductors can also serve as separators to prevent short circuits between the two electrodes. PVA‐based proton‐rich gel electrolyte is widely used due to its high chemical stability, ionic conductivity, expansive potential windows, low toxicity, and low cost.^[^ ^153^ ^]^

**Figure 15:**
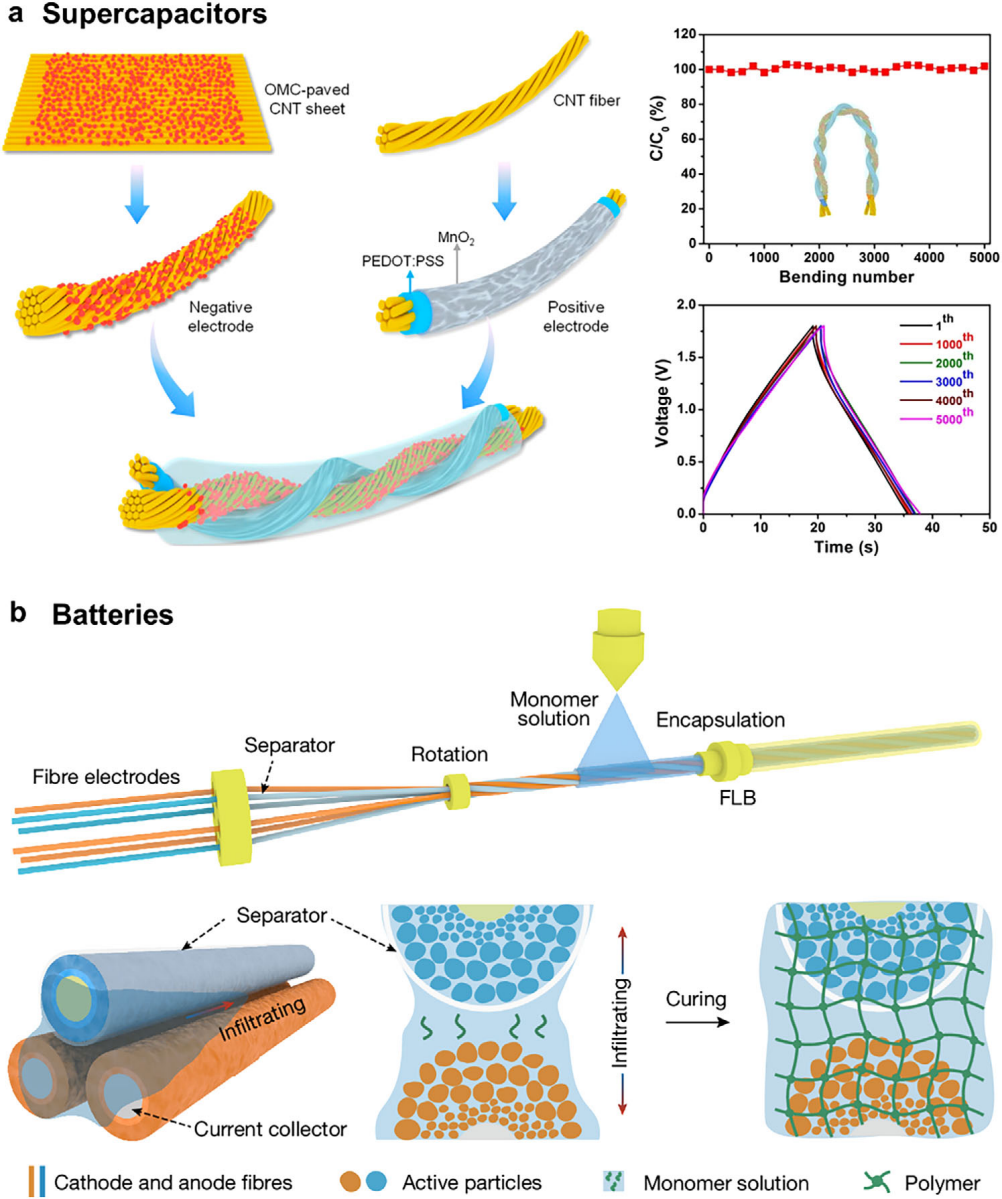
a) Schematic illustration to the fabrication of fiber‐shaped asymmetric supercapacitor. Dependence of specific volumetric capacitance on bending cycle number. Charge and discharge curves after different bending cycles. Reproduced with permission.^[^ ^154^ ^]^ Copyright 2016. ACS. b) Schematic of the fabrication process of an FLB based on a polymer gel electrolyte. Infiltrating process of the monomer solution on the cathode and anode fibers through the aligned channels. Monomer solution sequentially infiltrating into the networked large and small channels, followed by polymerization to form the polymer gel electrolyte. Reproduced under the terms of the CC‐BY Creative Commons Attribution 4.0 International license (https://creativecommons.org/licenses/by/4.0/).^[^ ^155^ ^]^ Copyright 2024, published by Springer Nature.

Regarding the fiber structure, there are several basic configurations. The parallel configuration has two aligned electrodes immersed in the electrolyte and then packed by a plastic tube or polymer layer. The parallel structure is suitable when electrode diameters are relatively large (e.g., millimeter level). However, it has low stability during deformation as the two fiber electrodes may deform differently, risking electrolyte leakage and device damage. The twisting configuration involves twisting the two electrodes together and packing them with electrolytes. This configuration is more mechanically stable during deformation, reducing the risk of structural issues. For instance, Cheng et al.^[^ ^154^ ^]^ developed a fiber‐shaped supercapacitor with high volumetric energy densities. They designed an asymmetric configuration and integrated multiple functional components as electrodes and ionic conductive gels as the electrolyte (**Figure** **15**a). These fiber‐based asymmetric supercapacitors exhibited high flexibility, stable cycling performance, and good rate performance even under bending. The coaxial configuration can be seen as a variant of a planar supercapacitor. It consists of a core fiber electrode, a separator or solid/gel electrolyte, and an outer electrode layer. It provides a more efficient electrode interface and is structurally stable under deformation. However, continuously depositing capacitive materials on a coaxial‐fiber electrode is challenging, limiting its large‐scale application.

#### 5.3.2 Batteries

Ionic conductors, or electrolytes, serve as the medium that enables the movement of ions between the anode and the cathode during the charge and discharge processes of batteries. The ion transfer is fundamental for sustaining the electrochemical reactions that generate and store electrical energy. A high‐quality ionic conductor in a fiber battery must form a stable and low‐resistance interface with the electrodes. This ensures efficient charge transfer, which significantly impacts the overall performance of the battery, including its capacity, voltage output, and cycle life. For example, Lu et al.^[^ ^155^ ^]^ reported a strategy for designing electrode channel structures in high‐performance wearable batteries. The polymer gel electrolytes formed intimate and stable interfaces with electrodes by infiltrating the monomer solution along the aligned channels and then into the networked channels, followed by the in‐situ polymerization. The resulting fiber lithium‐ion battery demonstrated high electrochemical performances, such as an energy density of around 128 Wh kg^−1^. This strategy also allowed for the production of high‐rate fiber lithium‐ion batteries (3600 m h^−1^ per winding unit). The batteries were woven into a 50 cm × 30 cm textile, providing an output capacity of 2975 mAh.

The design of gel electrolytes focuses on optimizing the polymer network structure to ensure uniform distribution of the liquid component and efficient ion transport pathways. This careful design of ionic conductors in fiber batteries is essential for unlocking their full potential in various applications, from wearable electronics to flexible energy storage systems.

### 5.4 Ionic Processors

In electronic devices, information processing, transmission and storage functions rely on the transport of electrons. In contrast, these functions are predominantly realized by ions in biological systems. The difference in information carriers between electronic devices and organisms causes a signal mismatch when designing bioelectronic interfaces for healthcare and human‐machine interaction. To overcome this hurdle, ionotronics that integrate ion transport and electron transfer have been utilized as transducers between ionic and electronic circuits. In particular, fiber‐based ionic processors have emerged as a promising alternative for bioelectrical information collection and processing, offering unique advantages such as miniaturization, stable contact interface with biological tissues, and potential integration into wearable and bio‐compatible devices.^[^ ^156^ ^]^ This part focuses on four key components of fiber‐based ionic processors: ionic diodes, ionic transistors, and ionic transmitters.

#### 5.4.1 Ionic Diodes

Ionic diodes are fundamental building blocks in ionic processors, enabling the rectification and switching of ionic currents. Fiber‐based ionic diodes have shown remarkable potential due to their unique structural and functional properties. For instance, Xing et al.^[^ ^157^ ^]^ employed an integrated opposite charge grafting method to design a polyelectrolyte‐based ionic‐junction fiber. The polycation/polyanion heterojunction induced the formation of an ionic double layer (IDL), mimicking the depletion region in a semiconducting P‐N junction. Under a reverse bias electric field, mobile Na^+^ and Cl^−^ ions moved away from the interface. The resulting narrow depletion area led to a “cutoff” state, preventing current flow. Conversely, the diode entered an “open” state to permit current flow. The prepared fiber‐shaped ionic diode achieved a rectification ratio of 30 at ± 3 V.

**Figure 16:**
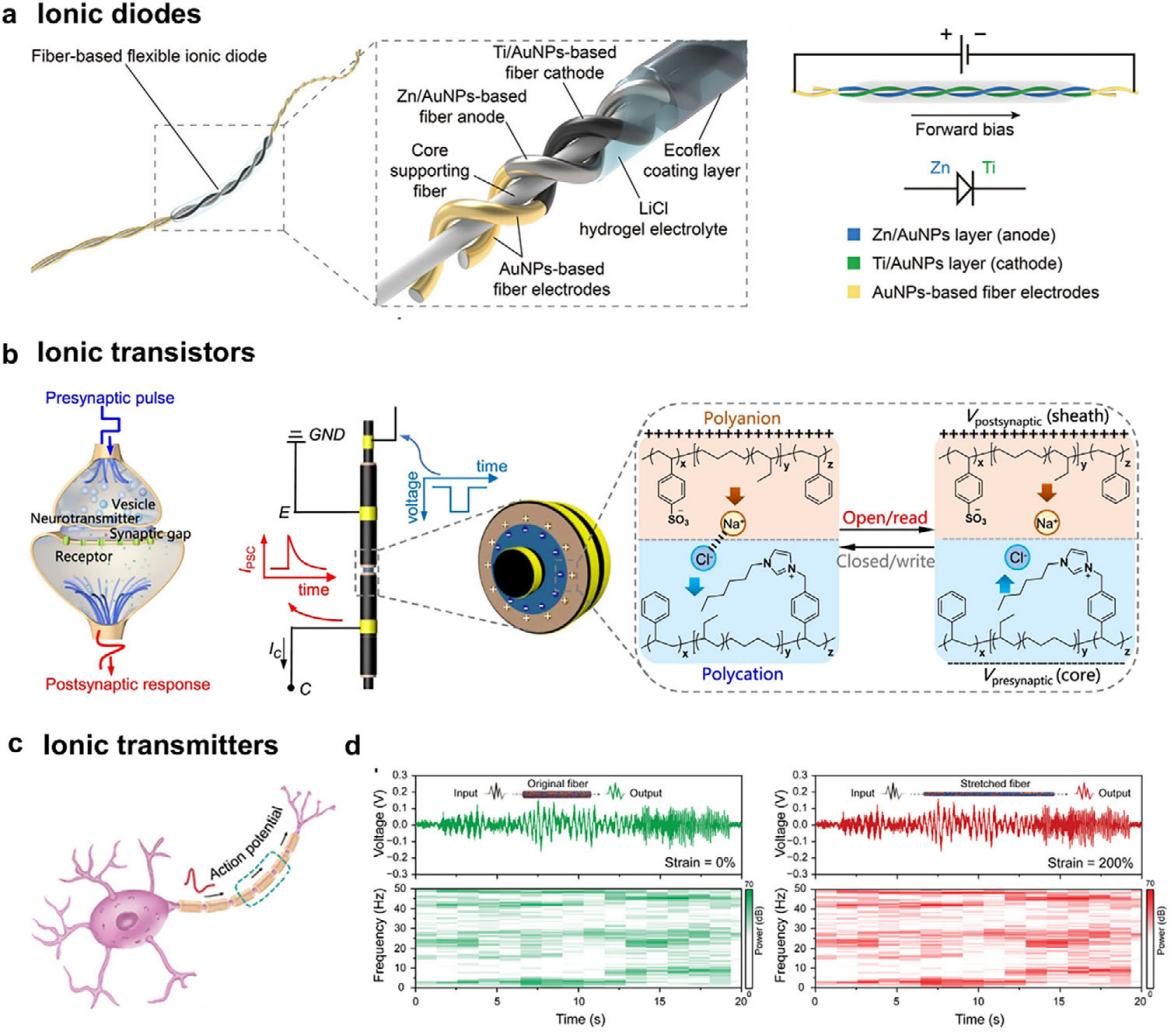
a) Schematic illustration depicting the structure of the fiber‐based flexible ionic diode and the simplified circuit diagram of the fiber‐based flexible ionic diode. Reproduced under the terms of the CC‐BY Creative Commons Attribution 4.0 International license (https://creativecommons.org/licenses/by/4.0/).^[^ ^158^ ^]^ Copyright 2024, published by Wiley‐VCH. b) Schematic illustration of a natural biological synapse and the three‐terminal synaptic IBJT. A negative presynaptic potential (V_presynaptic_) drove Cl^−^ into the postsynaptic electrode while Na^+^ accumulated near the heterojunction in the device due to the application of the postsynaptic voltage (V_postsynaptic_) during an open‐read operation. Reproduced under the terms of the CC‐BY Creative Commons Attribution 4.0 International license (https://creativecommons.org/licenses/by/4.0/).^[^ ^157^ ^]^ Copyright 2023, published by Springer Nature. c) Diagram about the myelinated axon's structure. The neural signal travels along the myelinated axon in the form of the action potential. Reproduced with permission. Copyright 2022.^[^ ^126^ ^]^ Wiley‐VCH. d) Transmitted sound wave signals and corresponding FFT spectrograms through the bicontinuous fiber at 0% and 200% strains, respectively. Reproduced with permission.^[^ ^80^ ^]^ Copyright 2024. Wiley‐VCH.

To reach the rectification ratio level of planar electronic counterparts (10^3^–10^6^), Woo et al.^[^ ^158^ ^]^ presented a fiber‐based ionic diode with high robustness and a rectification ratio of 2773 (**Figure** **16**a). The diode is composed of a double helical Zn‐based fiber anode, a Ti‐based fiber cathode on Au nanoparticle‐based flexible fiber electrodes, and a LiCl hydrogel electrolyte. The current rectification performance of the diode was realized by the redox reactions combined with ion transportation through the hydrogel electrolyte.

#### 5.4.2 Ionic Transistors

Ionic transistors play a vital role in controlling and amplifying ionic signals in fiber‐shaped ionic processors. One example is a fiber‐shaped ionic transistor that utilizes a gel electrolyte as the conducting medium. The operation of transistors is based on the modulation of the ion doping into the semiconductor channel from the ionic conductor by an applied gate voltage. When a gate voltage is applied, the ions in the electrolyte are redistributed, leading to a change in the conductivity of the channel region by doping. This modulation effect allows for the amplification and switching of ionic signals, similar to the operation of traditional electronic transistors. For instance, Kim et al.^[^ ^159^ ^]^ developed fibrous organic electrochemical transistors. These transistors consist of a double‐stranded assembly of electrode microfibers and an ionogel gate insulator. A practical textile artificial neural network system was demonstrated by an array composed of 100 distinct synapses, weaving 10 neurofibers and 10 gate‐microfibers. The transfer characteristics of the 10 transistors exhibited a uniform memory window.

On the basis of distinct short or long drain‐current characteristics induced by ions penetration into the active channels, synaptic functionalities such as short‐term and long‐term plasticity, including paired‐pulse facilitation (PPF), excitatory postsynaptic current (EPSC), and inhibitory postsynaptic current (IPSC), can be achieved. Xing et al.^[^ ^157^ ^]^ reported a transistor that can exhibit long‐term plasticity (LTP) by retaining stored data from trapped ions, enabling it to remember previous activity. In the fiber‐shaped device architecture, the CNT fiber serves as the axon of a presynaptic neuron. It transfers the presynaptic pulse to the post‐neuron, converting it into the corresponding IPSC via Na^+^ and Cl^−^ transport (Figure 16b). The Ionic synaptic transistors are akin to a series connection of two two‐terminal fiber‐shaped ionic diodes. The fiber‐based transistors enable its integration into flexible and wearable circuits, opening up new possibilities for bio‐sensing and neuromorphic computing applications.

#### 5.4.3 Ionic Transmitters

Ionic transmitters, mimicking mammalian nervous system axons, handle ionic information transmission in biological systems. Compared to electrical wires, ionic conductors, with their low operating voltage, reduced energy consumption, and favorable biointerface, can be used as interconnects for high‐fidelity signal transmission.^[^ ^160^ ^]^

Two strategies are used to construct ionic transmitters. One involves sandwiching a dielectric elastomer between two ionic conductors to mimic the functionality of an axon by forming electrical double layers for signal transmission (Figure 16c).^[^ ^126^ ^]^ The other focuses on developing strain‐insensitive ionic conductors. For example, Ye et al.^[^ ^80^ ^]^ fabricated ionic conductive fibers with a solid‐liquid bicontinuous microstructure. These fibers maintained highly stable ionic conduction, transmitting high‐fidelity audio signals, and showed only a 7% resistance increase at 200% strain (Figure 16d).

Despite these achievements, the applications of ionic transmitters in electrical signal transmission remain limited. In flexible bio‐sensing devices, for instance, real‐time monitoring and analysis of biological signals from tissue and nerve have yet to be realized.

## 6 Challenges and Perspectives

**Figure 17:**
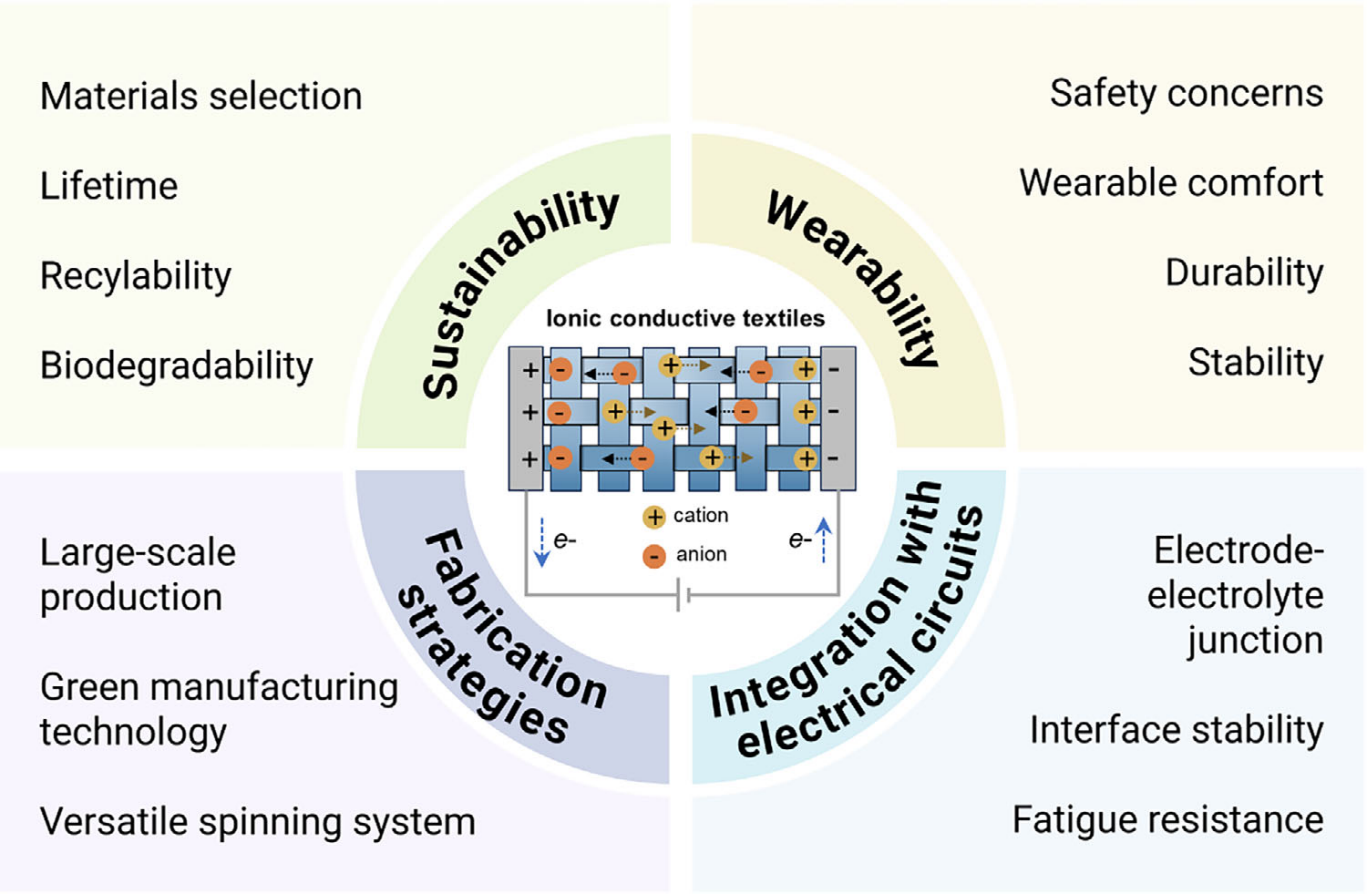
Challenges and perspectives for the design of the next generation of ionic conductive textiles.

Although ionic conductive textiles exhibited excellent performance in wearable devices, there are still challenges regarding sustainability, wearability, fabrication strategies, and integration with electronic systems (**Figure** **17**). We highlight the challenges and perspectives in the design and development of next‐generation ionic conductive textiles in this session.

### 6.1 Sustainability

In recent years, there has been a growing and intense focus on the sustainability of wearable fabrics. As environmental concerns take center stage globally, consumers and industries are becoming more conscious of the ecological footprint of products, and wearable devices are no exception. In terms of materials selection of polymer matrix and electrolytes, the current options often rely on non‐renewable resources and may involve the use of potentially harmful chemicals. Many traditional polymer matrices are petroleum‐based, which not only depletes finite resources but also has a significant carbon footprint.^[^ ^161^ ^]^ Electrolytes, containing salts that may not be environmentally friendly or bio‐friendly, cause waste pollution and skin inflammation. This poses a threat to the long‐term viability and ecological impact of these materials. To address this, a shift towards bio‐based and sustainable polymer matrices is essential by exploring the use of natural polymers like cellulose, chitosan, and alginate as renewable alternatives.^[^ ^162^ ^]^ These materials can be sourced from abundant natural resources and have the potential to reduce the environmental burden. Additionally, developing electrolytes from non‐toxic and biodegradable components, such as ionic liquids derived from natural sources or solid‐state electrolytes with environmentally friendly compositions, can enhance the sustainability of the overall system. However, the high cost of ionic liquids and electrolytes (e.g., LiTFSI) presents a major obstacle to large‐scale production and commercialization. To achieve sustainability, environmentally friendly and cost‐effective materials must be adopted for fabricating ionic conductive textiles.

The lifetime of ionic conductive textiles is another concern. These materials are often subjected to various mechanical stresses and environmental factors during use, which can lead to degradation and a decrease in performance over time. Repeated bending, stretching, and washing can damage the conductive pathways within the fibers/textiles, reducing their ionic conductivity and functionality over time, which compromises cost‐per‐use efficiency. To prolong service life, advanced material engineering techniques can be employed, including the development of hybrid materials that combine the strength and flexibility of different polymers, and the incorporation of dynamic bonds (e.g., hydrogen bonds, boronic esters) to enhance mechanical properties and self‐healing performance, therefore extending lifespan.^[^ ^163^, ^164^ ^]^ Surface modifications and coatings can also be applied to protect the conductive components from environmental degradation.^[^ ^165^ ^]^

Recyclability and biodegradability represent critical sustainability considerations for ionic conductive fibers and textiles, offering benefits of reducing both material expenses and waste management costs. Ionic conductive textiles fabricated by petroleum‐based materials are difficult to recycle due to their complex compositions and the integration of different components (e.g., cotton and spandex). To overcome these challenges, designing materials with a modular architecture that allows for easy separation and recovery of the different components during recycling is a promising approach, which involves the use of reversible chemical bonds or physical interactions that can be broken down under specific conditions, such as light, heat, pH, and ultrasound.^[^ ^166^, ^167^ ^]^ Another strategy is to fabricate ionic conductive textiles by using biopolymers such as cellulose, silk, and cotton, which can be recycled by mechanical pulverization and chemical solvent treatment.

Moreover, the materials for ionic conductors can accumulate in the environment and cause pollution if they are not biodegradable. Developing biodegradable materials that can naturally decompose in the environment without leaving harmful residues is of great importance. During the process of biodegradation, the ionic conductors are broken down into simpler compounds (such as water, carbon dioxide, salts, and other small molecules) through the action of microorganisms (such as bacteria, fungi, etc.), thus reducing the long‐term pollution of organic waste to the environment.^[^ ^91^, ^168^, ^169^ ^]^

### 6.2 Wearability

Ionic conductive textiles that come into direct contact with the skin should meet the requirements of wearability, including safety concerns, wearable comfort, durability, and stability. Biocompatibility is the most important factor when considering safety issues. Biocompatibility encompasses multiple aspects, including non‐toxicity, non‐immunogenicity, and the ability to interact favorably with biological tissues.^[^ ^170^ ^]^ Ensuring biocompatibility is essential to prevent adverse reactions such as inflammation, allergic responses, or tissue damage, which could potentially impede the wound‐healing process rather than facilitate it. Some of the materials used in ionic conductive textiles may not be inherently biocompatible, which can pose a risk to the health of wearers. Selecting materials that are intrinsically biocompatible or modifying the surface of the materials to improve their biocompatibility can be effective strategies. For example, surface functionalization with biomolecules or the use of biocompatible coatings can reduce the potential for immune responses and improve compatibility with the skin. After achieving excellent biocompatibility, electrical stimulation can be applied through ionic textiles to accelerate wound healing to improve wearable safety further.^[^ ^20^ ^]^

Wearable comfort is a key consideration for the practical application of ionic conductive textiles. Permeability is an important factor as these materials need to allow the passage of air and moisture to maintain a comfortable microclimate on the skin. The use of electrospun nanofibers or hierarchical textile structure design provides a large surface area for gas and moisture exchange while maintaining the necessary conductivity.^[^ ^6^, ^171^ ^]^ Achieving a lightweight design is also crucial for wearable comfort. Heavy or bulky ionic conductive fibers/textiles can cause discomfort and restrict movement. The current materials and manufacturing processes may result in relatively thick fibers (≈1000 µm), which limit their application in wearable devices. To address this problem, lightweight materials and advanced fabrication methods can be explored. For instance, using ultrathin conductive coatings or incorporating conductive nanoparticles in a lightweight polymer matrix can reduce the overall weight of the fibers/textiles.^[^ ^143^ ^]^

Textiles often face mechanical stress and friction during daily use, such as stretching, bending, and rubbing. Therefore, durability is crucial for ionic conductive textiles for long‐term wear.^[^ ^172^ ^]^ Notably, their conductive components must retain integrity and conductivity through multiple deformation cycles. The leakage of liquid electrolytes in the polymer matrix causes conductivity loss under repeated stress, limiting the lifespan of the textiles. To improve durability, research should focus on developing more stable ionic conductive materials and better integration methods. Incorporating liquid‐free ionoelastomers with high mechanical strength or utilizing polymers with functional groups to enhance the adhesive interactions between conductive ions and the non‐conductive matrix ensures that the ionic conductive textiles can withstand the rigors of daily wear and tear while maintaining their conductive functionality.

Additionally, ionic conductive textiles must be stable in various environments and usage scenarios. They should be able to function effectively in varying temperatures, humidity levels, and mechanical conditions. For example, in high‐humidity environments, the conductivities of the textiles may be affected due to moisture absorption. Under low temperatures, the flexibility and conductivity of ionic conductors will be compromised due to freezing problems. To enhance stability, materials with excellent anti‐freezing and anti‐dehydration performances that maintain high flexibility and ionic conductivity, like eutectogel, ionoelastomer, and poly(ionic liquids), could be developed. Also, smart materials that can adjust their conductivity or mechanical properties in response to environmental changes, such as thermoresponsive or hygroscopic polymers, could be incorporated into the textile structure.^[^ ^173^ ^]^ Additionally, designing textiles with modular components that can be easily disintegrated or replaced according to different usage requirements can improve their adaptability.^[^ ^174^ ^]^

### 6.3 Fabrication Strategies

The fabrication of ionic conductive textiles at a large scale is a significant challenge in fiber spinning and textile manufacturing. For fiber spinning, the integration of advanced spinning technologies compatible with high‐speed fabrication, such as draw spinning, electrospinning, and wet spinning, with high‐throughput production lines, can be involved in large‐scale production. However, wet spinning requires a high volume of coagulation baths, and dry spinning requires precise environmental control (e.g., humidity, temperature), which largely increases manufacturing costs. Current handicraft manufacturing methods are limited in terms of production efficiency, making it difficult to meet the growing demand for ionic conductive textiles. Scaling up the production processes often leads to issues such as inconsistent quality, higher production costs, and difficulties in maintaining the desired properties of the textiles. Continuous and automated manufacturing processes need to be developed to enable large‐scale production. Additionally, optimizing the process parameters and developing quality control systems to ensure the uniformity and reproducibility of the ionic conductive textiles during large‐scale production are essential.

Green manufacturing technology has aroused the attention of society during the fabrication of ionic conductive textiles. Traditional manufacturing processes may involve the use of large amounts of solvents, energy‐intensive procedures, and the generation of byproduct wastes, which harm the environment. To reduce waste, the development of solvent‐free or low‐solvent manufacturing methods, such as dry spinning or 3D printing techniques, can be explored.^[^ ^161^ ^]^ These methods can reduce the environmental footprint and improve the sustainability of the production process. In addition, the use of renewable energy sources and the implementation of waste management strategies to recycle and reuse materials and by‐products can further enhance the green manufacturing of these materials.

A versatile spinning approach is needed to fabricate ionic conductive textiles with different properties and structures. The current spinning methods may be limited in their ability to produce fibers with specific morphologies, compositions, or functionalities. To overcome this limitation, the development of hybrid spinning techniques that combine the advantages of different spinning methods can be considered. For example, combining electrospinning with melt spinning or wet spinning to produce fibers with complex structures and tailored properties.^[^ ^175^ ^]^ Moreover, the use of in‐situ polymerization or functionalization during the spinning process can enable the incorporation of conductive components and other functional materials directly into the fibers, expanding the range of possible applications.

### 6.4 Integration with Electronic Systems

As the demand for flexible and wearable electronics continues to grow, exploring the integration between ionic conductive textiles and electronic systems becomes crucial for creating seamless, functional, and innovative ionotronic systems that can be comfortably incorporated into daily life. The electric double layer (EDL) formed at the electrode‐electrolyte interface is the main driving force for the operation of ionic devices and presents several challenges.^[^ ^176^ ^]^ One of the key issues is electrode‐electrolyte junction sensing. Poor contact between ionic conductors and metal electrodes (e.g., Au, Ag) leads to high impedance, degrading signal transmission. The interface between the electrodes and the ionic conductive materials needs to be carefully designed to ensure efficient charge transfer and accurate sensing. The development of electrode materials with enhanced compatibility with ionic conductors, such as using carbon‐based electrodes with large area‐to‐volume ratio (e.g., carbon nanotube, graphene) and conductive polymer electrodes (e.g., PEDOT:PSS) with porous structure to reduce interfacial resistance, could improve the sensitivity of electrode‐electrolyte junction sensing.^[^ ^177^, ^178^ ^]^ Additionally, surface modifications and functionalization of the electrodes can improve the wettability, conformability, and adhesion of the electrolyte, facilitating better charge transfer for supercapacitors.^[^ ^179^ ^]^

The stability of the interface between electronic and ionic conductors is another critical challenge. The different chemical and physical properties of electronic and ionic conductors can lead to interfacial instabilities, such as the formation of unwanted chemical reactions or the degradation of the interface over time, affecting the performance and reliability of the integrated devices.^[^ ^180^ ^]^ Apart from the abovementioned selection and modification of electrodes, the ionic conductors can also be designed for a broader electrochemical window to avoid redox reactions at the electrode‐electrolyte interfaces. Using liquid‐free ionic conductors as electrolytes is a promising approach to broaden the electrochemical windows while allowing for efficient charge transfer due to the absence of liquid.^[^ ^59^ ^]^ Additionally, optimizing the fabrication processes to ensure a clean and defect‐free interface can also contribute to improved stability.

The fatigue resistance and compatibility of devices when integrating ionic conductive textiles with existing electronic systems is a significant concern. During operation, these devices are subjected to repeated mechanical deformations, which can cause damage to the interface between the ionic conductive devices and the electrical circuits, leading to a decrease in performance and eventually device failure. Increasing the interfacial adhesion is effective in improving fatigue resistance since the strain is distributed to flexible and stretchable ionic conductors instead of rigid electrical wires.^[^ ^181^ ^]^ Additionally, encapsulation and packaging techniques can be employed to protect the interface and the device from environmental factors and mechanical stress, enhancing the overall fatigue resistance.^[^ ^182^ ^]^ Another strategy is to develop stretchable electrical circuits, such as electrically conductive polymers or stretchable metal nanowires.^[^ ^183^, ^184^ ^]^ Besides, the problem of signal frequency mismatch also exists since ionic conductors operate at low frequencies (<1 kHz), whereas conventional electronics require high‐frequency signals (>1 MHz), complicating direct coupling, especially in bioelectronics for physiological signal monitoring. Integrating impedance‐matching circuits (e.g., voltage amplifiers) is a promising method to bridge ionic‐electronic signal gaps.^[^ ^185^, ^186^ ^]^

In conclusion, the development of ionic conductive fibers/textiles holds great potential for a wide range of applications. However, addressing the challenges related to sustainability, wearable comfort, fabrication technology, and integration with electronic systems is crucial for their successful implementation. By implementing the proposed solution strategies and continuing to explore new materials and technologies, significant progress can be made in the field of ionic conductive textiles.

## 7 Conclusion

In this review, we focus on the recent development of ionic conductive textiles for wearable technology. Soft ionic conductors, with skin‐like flexibility and tissue‐like ion dynamics, are highly suitable for intimate applications in wearable electronics, ionotronics for sensing, energy harvesting, energy storage, signal transmission, and bioelectronics. Transforming ionic conductors into fiber and textile forms, instead of relying on rigid electron‐based conductors, marks a revolutionary technology in wearable electronics, offering benefits in device‐human compatibility, wearing comfort, and sustainability. Here, we summarize the key characteristics of ionic conductors, fabrication methods for ionic conductive fibers, yarns, and textiles, performance metrics, and diverse applications of these fiber‐based ionic conductors. By discussing perspectives and potential challenges regarding the future design and development of fiber‐based ionic conductors in terms of sustainability, wearable comfort, fabrication technology, and integration with electrical systems, we aim to highlight the potential of fiber‐based ionic conductors as crucial components for next‐generation smart textiles in the applications of personalized healthcare, power supply, bioelectrical signal transmission, and intelligent interactive clothing. In the future, it is foreseen that significant progress will be made in developing high‐performance and conformable ionic conductive textiles using sustainable materials and large‐scale fabrication strategies.

## Conflict of Interest

The authors declare no conflict of interest.
